# SIRT1 deacetylates GAPDH to drive microglial glycolysis and neuroinflammation

**DOI:** 10.3389/fimmu.2026.1883095

**Published:** 2026-08-18

**Authors:** Chou-Yi Hsu, Ibrokhim Sapaev, Ozodbek Nematov, Rustam Turakulov, Sarmad S. Abdullah, Zeina T. Khaleel, Enas R. Alwaily, Majid S. Jabir

**Affiliations:** 1 Innovation Department, Yuan An Bioresearch & Technology Co., Ltd., Tainan, Taiwan; 2 Tashkent Institute of Irrigation and Agricultural Mechanization Engineers, National Research University, Tashkent, Uzbekistan; 3 School of Engineering, Central Asian University, Tashkent, Uzbekistan; 4 Western Caspian University, Scientific researcher, Baku, Azerbaijan; 5 Jizzakh State Pedagogical University, Deparment of General History, Jizzakh, Uzbekistan; 6 Department of Internal Medicine and Family, Tashkent State Medical University, Tashkent, Uzbekistan; 7 College of Pharmacy Al- Mustafa University, Baghdad, Iraq; 8 College of Sciences, Al-Ayen Iraqi University, An Nasiriyah, Iraq; 9 College of Applied Sciences, University of Technology, Baghdad, Iraq

**Keywords:** glyceraldehyde-3-phosphate dehydrogenase, glycolysis, lysine acetylation, microglia, neuroinflammation, Sirtuin 1

## Abstract

**Introduction:**

Microglial activation drives neuroinflammation through a metabolic switch from oxidative phosphorylation to aerobic glycolysis; however, the molecular mechanisms governing this transition remain poorly defined. Glyceraldehyde-3-phosphate dehydrogenase (GAPDH), sirtuin 1 (SIRT1), lipopolysaccharide (LPS), and interferon-gamma (IFN-γ) are central to this study; GAPDH plays plays a key regulatory role in this switch, and its activity is modulated by reversible acetylation at lysine 254 (K254). It remains unclear whether sirtuin deacetylases regulate this modification in microglia.

**Methods:**

Here, we demonstrate that SIRT1 physically associates with GAPDH in murine microglia and deacetylates K254 under basal conditions. Inflammatory activation using LPS/IFN-γ reduced SIRT1 protein levels and deacetylase activity by approximately 50%, leading to a 2.5-fold increase in K254 acetylation. Pharmacological activation of SIRT1 (SRT1720) reversed this modification and enhanced glycolytic output, mimicking the effects of the deacetylation-mimetic K254R mutant. To isolate the causal role of K254, we replaced endogenous GAPDH with K254R or acetylation-mimetic (K254Q) mutant proteins.

**Results:**

K254R microglia exhibited approximately 35% higher GAPDH enzymatic activity, 40% greater glycolytic flux, and 1.6- to 2.2-fold higher secretion of TNF-α, IL-1β, IL-6, and IL-12p70 than K254Q cells. Glycolytic inhibition with 2-deoxyglucose reduced most of the excess cytokines, confirming enhanced flux as the causal factor in K254-driven inflammatory amplification.

**Discussion:**

Thus, SIRT1–GAPDH signaling represents a post-translational axis linking sirtuin activity directly to glycolytic enzyme function, distinct from SIRT1's traditional transcriptional roles and serving as a viable molecular checkpoint in microglial immunometabolism.

## 1 Introduction

Microglia are the brain’s primary first responders, acting as immune sentinels involved in the development of nearly every chronic neurological disease, from Alzheimer’s and Parkinson’s diseases to multiple sclerosis and clinical depression. Interestingly, the drivers of microglial dysfunction are fundamentally metabolic in nature. When these cells shift from a stable “homeostatic” state to a pro-inflammatory one, they completely change their energy production, moving from oxidative phosphorylation to aerobic glycolysis. This metabolic shift closely resembles the “Warburg-like” effect observed in peripheral immune cells, such as T cells (1, 2). We now know that bioenergetic reprogramming is not merely a side effect of microglial activation. Instead, the intensity of glycolytic flux dictates cytokine release, the ability of cells to clear debris, and the duration of the inflammatory response (3–5). Understanding the triggers and sustainers of this metabolic “switch” is essential for developing effective anti-inflammatory therapies. Within this glycolytic program, GAPDH has emerged as a crucial player. Once dismissed as a mundane “housekeeping” enzyme, it is now recognized as a highly regulated “moonlighting” protein influenced by various post-translational modifications. (6, 7).

In microglia, GAPDH is at the top of the inflammatory metabolic hierarchy. It not only processes glucose but also regulates cell polarization by controlling IFN-γ expression and directing traffic to the serine biosynthesis pathway (3) Moreover, when GAPDH enters the nucleus of cortical microglia, it can drive cognitive “stiffness” or inflexibility under stress (8). Essentially, GAPDH serves as a crossroads where the metabolic state and immune behavior of a cell converge.

However, the regulation of GAPDH at this crossroads remains unclear. The enzyme undergoes several modifications, including S-nitrosylation, oxidation, and reversible acetylation. Acetylation at K254 directly enhances its activity and promotes aerobic glycolysis. While this mechanism was initially discovered in liver cells and recently linked to T cell inflammation (9). This question is important because acetyl-lysine “marks” are not permanent; they are removed by a small druggable family of enzymes known as sirtuins. Among these, SIRT1 is notable for its role in maintaining brain health. SIRT1 levels naturally decline with age or nicotinamide adenine dinucleotide (NAD_+_) depletion, a decrease that correlates with “primed” or hyperreactive microglia in the aging brain (10). Historically, SIRT1 has been known to suppress microglial activation by stabilizing mitochondria and suppressing inflammatory pathways (3, 11, 12), However, recent evidence suggests that SIRT1 does more than merely act as a genetic brake; it may also directly regulate metabolic enzymes, such as by dampening lactate production in models of Parkinson’s disease (PD) (13, 14). In this study, we investigated whether SIRT1 serves as a specific “eraser” for GAPDH acetylation at lysine 254 in microglia, thereby inhibiting runaway glycolysis and neuroinflammation. Using activated primary microglia, pharmacological tools, CRISPR-ready mutants, and Seahorse flux analysis, we mapped a specific molecular checkpoint. This site represents a promising target for therapeutic intervention in chronic neurological diseases.

## 2 Materials and methods

### 2.1 Cell culture and reagents

BV-2 murine microglia (ATCC) and primary microglia isolated from postnatal day 3–5 C57BL/6J pups were used in this study. BV-2 cells were maintained in DMEM supplemented with 10% FBS, 2 mM L-glutamine, and 1% penicillin-streptomycin at 37 °C in a 5% CO_2_ atmosphere. Primary microglia were isolated using anti-CD11b magnetic bead separation (Miltenyi Biotec) following meningeal stripping and cultured in DMEM/F-12 (1:1) with 10% FBS and 10 ng/mL M-CSF for 7 days. Microglial activation, referred to as “LPS/IFN-γ stimulation,” was induced with 100 ng/mL LPS (E. coli O111:B4, Sigma-Aldrich) and 10 ng/mL IFN-γ (PeproTech) for 6 or 24 h. SIRT1 was pharmacologically modulated using the activator SRT1720 (5 μM; Cayman Chemical) or the selective inhibitor EX-527 (10 μM; Sigma-Aldrich), administered 1 hour before stimulation; dimethyl sulfoxide (DMSO; ≤0.1%) served as the vehicle. All experimental groups, treatments, and constructs are listed in **Table 1**.

**Table 1:** Experimental groups, pharmacological treatments, and genetic constructs.

Experimental group	Cell model	Treatment/construct	Dose & duration	Experimental purpose
Core control and inflammatory-stimulation groups				
Unstimulated control	BV-2; Primary microglia (C57BL/6J P3–5)	DMSO vehicle only (≤0.1% v/v)	—	Establish basal SIRT1 activity, K254 acetylation, and glycolytic parameters
wild-type (WT) GAPDH-FLAG	BV-2	pCMV-human-GAPDH-FLAG (WT); endogenous GAPDH suppressed by 3′ UTR siRNA	Replacement ≥75%; confirmed by anti-FLAG immunoblot	Intermediate control expressing endogenous-like acetylation mix
LPS/IFN-γ (6 h)	BV-2; Primary microglia	LPS (E. coli O111:B4, Sigma-Aldrich) + IFN-γ (PeproTech)	100 ng/mL LPS + 10 ng/mL IFN-γ for 6 h	Induce early-phase inflammatory activation; assess SIRT1 protein and deacetylase activity
LPS/IFN-γ (24 h)	BV-2; Primary microglia	LPS + IFN-γ (as above)	100 ng/mL LPS + 10 ng/mL IFN-γ for 24 h	Assess sustained inflammatory output: cytokines, NO, glycolytic flux, K254 acetylation
Pharmacological modulation groups				
SRT1720 + LPS/IFN-γ	BV-2; Primary microglia	SRT1720 (SIRT1 pharmacological activator; Cayman Chemical) pre-treatment + LPS/IFN-γ (24 h)	5 μM SRT1720; applied 1 h prior to stimulation	Restore SIRT1 activity; evaluate effects on K254 acetylation, glycoPER, and cytokine output
EX-527 + LPS/IFN-γ	BV-2; Primary microglia	EX-527 (selective SIRT1 inhibitor; Sigma-Aldrich) pre-treatment + LPS/IFN-γ (24 h)	10 μM EX-527; applied 1 h prior to stimulation	Further suppress SIRT1; serve as mechanistic counterpoint to SRT1720
Genetic manipulation and reconstitution groups				
SIRT1 siRNA	BV-2	ON-TARGETplus SMARTpool siRNA targeting SIRT1 (Dharmacon); Lipofectamine RNAiMAX	25 nM; 48 h before stimulation; knockdown >80%	Assess SIRT1 depletion at basal/early stimulation; not used as a late (24-h) loss-of-function validation
Non-targeting (NT) siRNA	BV-2	ON-TARGETplus NT control pool (Dharmacon)	25 nM; matched to SIRT1 siRNA timeline	siRNA control for off-target transfection effects
Endogenous GAPDH Suppression siRNA	BV-2	3′ UTR–targeting siRNA against murine GAPDH; 50 nM	50 nM; applied before mutant construct transfection	Suppress endogenous murine GAPDH to allow clean expression of human FLAG-tagged mutants
K254Q (acetylation-mimetic)	BV-2; Primary microglia	pCMV-human-GAPDH(K254Q)-FLAG; site-directed mutagenesis (QuikChange II XL, Agilent); Sanger confirmed	Endogenous GAPDH replaced (≥75%); expressed ± LPS/IFN-γ	Mimic constitutive K254 acetylation; reduce GAPDH catalytic activity; assess glycolytic and cytokine outputs
K254R (deacetylation-mimetic)	BV-2; Primary microglia	pCMV-human-GAPDH(K254R)-FLAG; site-directed mutagenesis; Sanger confirmed	Endogenous GAPDH replaced (≥75%); expressed ± LPS/IFN-γ	Mimic constitutive K254 deacetylation; enhance GAPDH catalytic activity; assess glycolytic and cytokine outputs
K254R + 2-DG	BV-2	K254R construct (as above) + 2-deoxyglucose (2-DG; Sigma-Aldrich) added before LPS/IFN-γ	5 mM 2-DG pre-treatment	Test causal requirement of enhanced glycolytic flux for pro-inflammatory amplification conferred by K254R
K254Q + 2-DG	BV-2	K254Q construct (as above) + 2-DG (5 mM)	5 mM 2-DG pre-treatment	Control for 2-DG effect in baseline-glycolysis (low-activity) condition

Summary of all experimental groups, interventions, and genetic constructs used in the study. All experiments performed in BV-2 murine microglia (American Type Culture Collection, ATCC) and primary microglia from postnatal day 3–5 C57BL/6J pups unless otherwise specified. n ≥ 3 independent biological replicates per group on separate days. LPS, lipopolysaccharide; IFN-γ, interferon-gamma; SIRT1, sirtuin 1; siRNA, small interfering RNA; NT, non-targeting; WTLAG, C-terminal FLAG epitope tag; DMSO, dimethyl sulfoxide; K254Q, glutamine substitution (acetylation-mimetic); K254R, arginine substitution (deacetylation-mimetic); 2-DG, 2-deoxyglucose; glycoPER, glycolytic proton efflux rate. References correspond to manuscript reference list.

### 2.2 Protein analysis and co-immunoprecipitation

**Figure 1:**
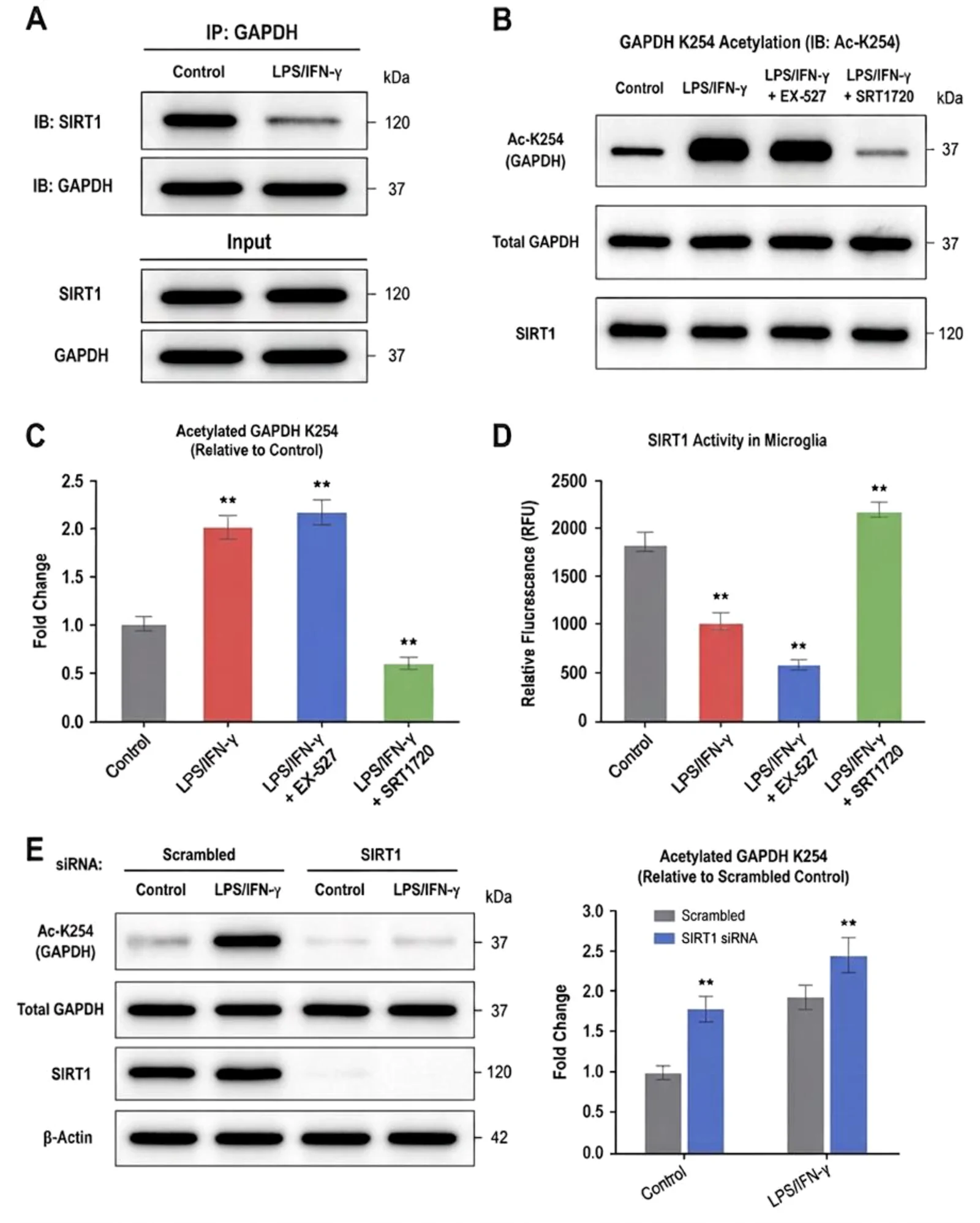
SIRT1 Co-Immunoprecipitates with GAPDH in Microglia and GAPDH K254 Acetylation is Regulated by SIRT1 Activity. BV-2 microglia were maintained under resting conditions or stimulated with LPS (100 ng mL_-_¹) plus IFN-γ (10 ng mL_-_¹) for 6 h, with or without SRT1720 (5 µM) or EX-527 (10 µM) (1-h pre-treatment). Lysates were processed for reciprocal co-immunoprecipitation (Co-IP) and site-specific immunoblotting. **(A)** Representative reciprocal Co-IP blots: anti-SIRT1 immunoprecipitates (clone D1D7) probed for endogenous GAPDH (clone 14C10) and vice versa, demonstrating a stable SIRT1–GAPDH interaction under basal conditions. **(B)** Densitometric quantification of co-precipitated GAPDH (SIRT1 pull-down) and SIRT1 (GAPDH pull-down) normalized to input. At this early time point, total SIRT1 in input lysates was not detectably changed; co-immunoprecipitated signals were therefore normalized to their respective immunoprecipitated bait, rather than interpreted as reflecting a difference in total SIRT1 abundance. **(C)** Site-specific immunoblot of immunoprecipitated GAPDH probed with anti-acetyl-GAPDH(K254) (Abcam ab280577), showing a robust increase in K254 acetylation after early LPS/IFN-γ stimulation. **(D)** Quantification of **(C)**, normalized to total immunoprecipitated GAPDH. K254 acetylation rose ~2.5-fold with stimulation, was attenuated by SRT1720 to levels indistinguishable from unstimulated controls (p = 0.18 vs. unstimulated; p < 0.01 vs. LPS/IFN-γ alone) and was further amplified by EX-527. **(E)** Pan-acetyl-lysine immunoblot (CST #9441) of whole-cell lysates, showing a broader rise in global acetylation upon stimulation; the K254 signal in **(D)** tracked SIRT1 activity more tightly than the global pattern, identifying K254 as a specific SIRT1-responsive mark. Data are mean ± SEM from n = 3–5 independent experiments. Co-IP stoichiometry comparisons used a paired two-tailed t-test; acetylation quantifications used one-way ANOVA with Tukey’s *post-hoc* test. *p < 0.05, **p < 0.01, ***p < 0.001; n.s., not significant. GAPDH, glyceraldehyde-3-phosphate dehydrogenase; SIRT1, sirtuin 1; IP, immunoprecipitation; IB, immunoblot; LPS, lipopolysaccharide; IFN-γ, interferon-gamma; SEM, standard error of the mean; ns, not significant; IgG, immunoglobulin G isotype control.

**Figure 2:**
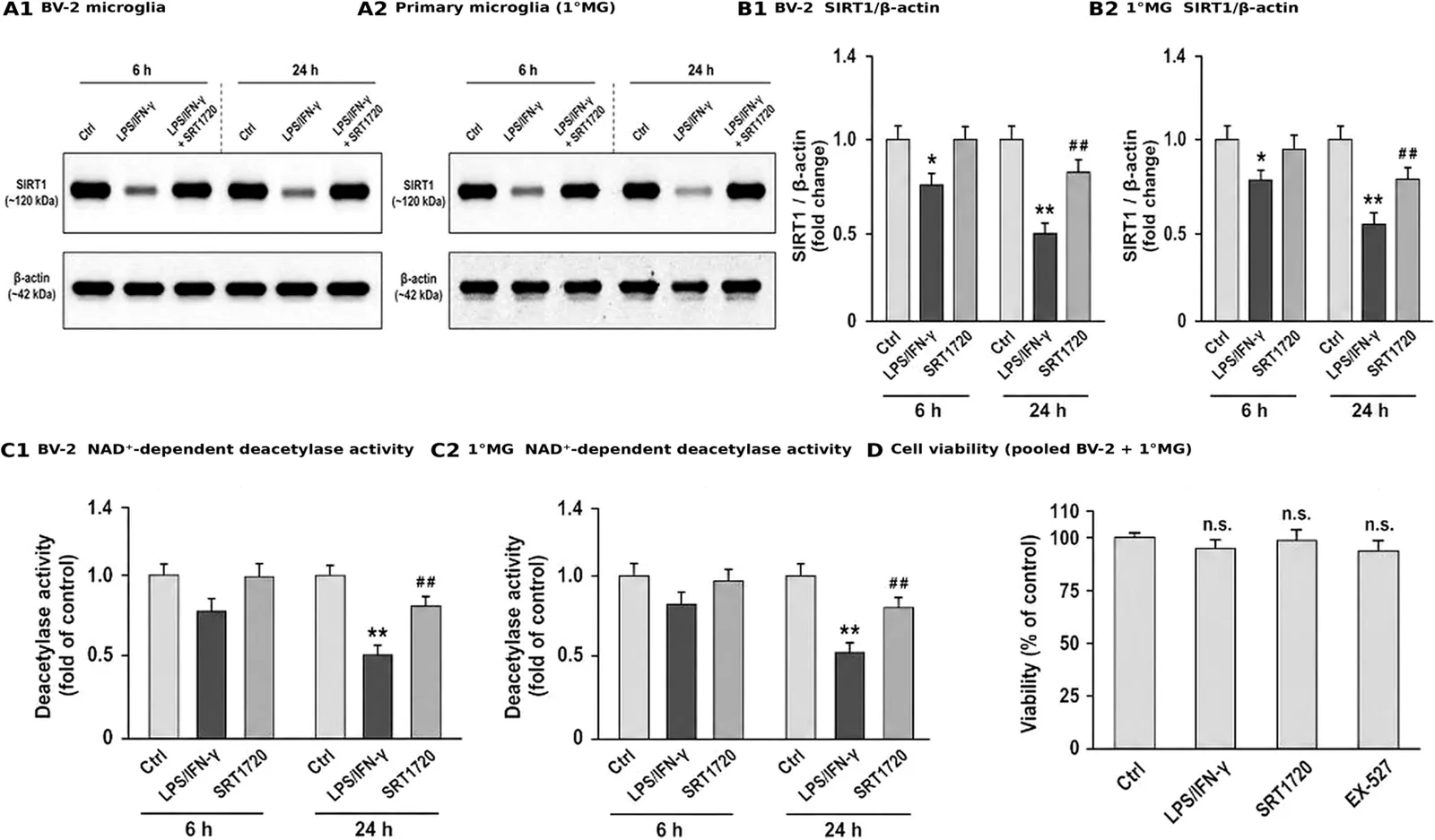
BV-2 murine microglia and primary microglia (isolated from postnatal day 3–5 C57BL/6J pups) were stimulated with LPS (100 ng mL_-_¹) and IFN-γ (10 ng mL_-_¹) for 6 or 24 hours. Parallel cohorts received a 1-hour pre-treatment with either the SIRT1 activator SRT1720 (5 µM) or the selective inhibitor EX-527 (10 µM), with DMSO (≤0.1%) serving as the vehicle control. Primary microglia were purified by CD11b_+_ magnetic bead separation (Miltenyi Biotec) after meningeal stripping and then cultured for 7 days in DMEM/F-12 (1:1) with 10% FBS and 10 ng mL_-_¹ M-CSF. Purity exceeded 95% Iba1_+_/CD68_+_ before stimulation began. Data for BV-2 and primary microglia (1°MG) are presented as adjacent sub-panels throughout to allow direct comparison between the two systems. **(A1, A2)** Representative immunoblots show total SIRT1 in whole-cell lysates from BV-2 (A1) and CD11b-purified primary microglia **(A2)** at 6 and 24 hours, with β-actin as a loading control. **(B1, B2)** Densitometric quantification, normalized to β-actin and expressed as fold change over untreated controls, shows that LPS/IFN-γ reduced SIRT1 to 0.48 ± 0.05-fold in BV-2 and 0.56 ± 0.07-fold in 1°MG by 24 hours. SRT1720 largely reversed this decline, restoring SIRT1 to 0.85 ± 0.04-fold in BV-2 and 0.82 ± 0.06-fold in 1°MG. In contrast, EX-527 further suppressed SIRT1 in both cell types. **(C1, C2)** NAD_+_-dependent deacetylase activity, measured fluorometrically in nuclear/cytoplasmic lysates, closely tracked protein levels: activity fell to approximately half of baseline by 24 hours in both BV-2 **(C1)** and 1°MG **(C2)**, confirming that the loss of SIRT1 protein translated into a corresponding loss of enzymatic function across both systems. **(D)** Cell viability, assessed by LDH release and Trypan blue exclusion and pooled across BV-2 and 1°MG, remained at or above 92% under all conditions. Therefore, the SIRT1 changes reported above cannot be attributed to cytotoxic effects of any treatment. Data are mean ± SEM from n = 4–5 independent biological replicates (mean ± SD where noted). Single-time-point comparisons used one-way ANOVA with Tukey’s *post-hoc* test; time-course comparisons used two-way ANOVA with Bonferroni correction. *p < 0.05, **p < 0.01 vs. Ctrl; ##p < 0.01 vs. LPS/IFN-γ; n.s., not significant. LPS, lipopolysaccharide; IFN-γ, interferon-gamma; SIRT1, sirtuin 1; SEM, standard error of the mean; LDH, lactate dehydrogenase; BV-2, murine microglial cell line; 1°MG, primary microglia; AU, arbitrary units; ns,non-significant.

Cells were lysed in RIPA buffer supplemented with protease inhibitors (Roche) and 10 mM nicotinamide to preserve acetylation. Protein concentrations were determined using a BCA assay (Thermo Fisher). Cleared lysates (500 μg) were incubated overnight at 4 °C with 2 μg of either anti-GAPDH (14C10, Cell Signaling Technology (CST)) or anti-SIRT1 (D1D7, CST), followed by a 2 h incubation with protein A/G magnetic beads (Millipore). The beads were washed three times, and bound proteins were eluted in SDS sample buffer, resolved by sodium dodecyl sulfate–polyacrylamide gel electrophoresis (SDS–PAGE), and transferred to polyvinylidene difluoride (PVDF) membranes. Membranes were probed for K254-acetylated GAPDH (Abcam ab280577), with total GAPDH, SIRT1, and pan-acetyl-lysine (CST #9441) serving as controls. Band intensities were quantified using ImageJ and normalized to the total immunoprecipitated protein. A comprehensive list of antibodies and reagents used is provided in **Table 2**. For the reciprocal co-immunoprecipitation and K254-acetylation analyses presented in **Figure 1**, cells were harvested after 6 hours of LPS/IFN-γ exposure. This early time point was chosen to evaluate the SIRT1–GAPDH complex prior to the substantial 24-h decrease in total SIRT1 observed in **Figure 2**. Input lanes, containing equal amounts of total lysate, were used to verify protein loading and immunoprecipitation specificity; they were not used for densitometric comparisons of 24-h SIRT1 abundance.

**Table 2:** Antibodies, assay kits, and key reagents.

Reagent/antibody	Target/application	Supplier	Catalog no.	Working conc./dilution
Anti-GAPDH (14C10)	GAPDH—Western blot (WB), Immunoprecipitation (IP)	CST	#2118	1:1000 (WB); 2 μg per 500 μg lysate (IP)
Anti-SIRT1 (D1D7)	SIRT1—WB, IP	Cell Signaling Technology	#9475	1:1000 (WB); 2 μg per 500 μg lysate (IP)
Anti-acetyl-GAPDH(K254)†	Site-specific K254 acetylation of GAPDH—WB (post-IP)	Abcam	ab280577	Manufacturer-recommended dilution; applied post-GAPDH IP
Pan-acetyl-lysine (AcK)‡	Global lysine acetylation—WB (whole-cell lysate specificity control)	Cell Signaling Technology	#9441	1:1000 (WB)
Anti-FLAG (M2)	C-terminal FLAG tag on transfected GAPDH constructs—WB (replacement efficiency verification)	Sigma-Aldrich	F1804	1:1000 (WB)
Anti-β-Actin	β-Actin—WB loading control	CST/Abcam	—	1:5000 (WB)
GAPDH Activity Assay Kit (colorimetric)	GAPDH enzymatic activity—NADH generation rate (nmol/min/μg protein)	Sigma-Aldrich	MAK277	Per manufacturer’s protocol; results in cell lysate
Seahorse Glycolytic Rate Assay Kit	Glycolytic proton efflux rate (glycoPER) and compensatory glycolysis—Seahorse XFe96	Agilent Technologies	103344-100	Sequential injection: Rotenone/Antimycin A then 2-DG; 5×10_4_ cells/well
Lactate Assay Kit (enzymatic)	Lactate concentration in conditioned medium; normalized to cell number (CyQUANT)	Sigma-Aldrich	MAK064	Per protocol; conditioned medium at 24 h
ProcartaPlex mouse inflammation panel (multiplex ELISA)	TNF-α, IL-1β, IL-6, IL-10, IL-12p70—conditioned medium protein quantification	Thermo Fisher Scientific	EPX050-20820-901	Samples in duplicate; MAGPIX instrument; 6-point standard curve
CyQUANT cell proliferation assay	Cell number normalization for metabolic assays	Thermo Fisher Scientific	C7026	Per manufacturer’s protocol
BCA protein assay kit	Protein quantification of cell lysates prior to WB and IP	Thermo Fisher Scientific	23225	Standard curve with BSA
RNeasy Mini kit	Total RNA extraction for qRT-PCR	Qiagen	74104	Per protocol; ≤30 mg starting material
iScript cDNA synthesis kit	Reverse transcription for qRT-PCR	Bio-Rad	1708890	Per protocol; 1 μg total RNA input
SRT1720 (SIRT1 activator)	Pharmacological SIRT1 activation—experimental modulator	Cayman Chemical	11085	5 μM; applied 1 h prior to LPS/IFN-γ stimulation
EX-527 (SIRT1 inhibitor, selective)	Pharmacological SIRT1 inhibition—mechanistic counterpoint	Sigma-Aldrich	E7034	10 μM; applied 1 h prior to LPS/IFN-γ stimulation
LPS (E. coli O111:B4)	Pro-inflammatory activation stimulus	Sigma-Aldrich	L4391	100 ng/mL; 6 h or 24 h
IFN-γ (recombinant murine)	Pro-inflammatory co-stimulus with LPS	PeproTech	315-05	10 ng/mL; 6 h or 24 h
2-Deoxyglucose (2-DG)	Glycolytic inhibitor—rescue experiments (**Figure 4** & **5**)	Sigma-Aldrich	D8375	5 mM; applied before LPS/IFN-γ stimulation
Protease inhibitor cocktail (complete)	Protect proteins during cell lysis; used with 10 mM nicotinamide (sirtuin inhibitor) at harvest	Roche	11836145001	1× per RIPA lysis buffer; harvested on ice
Lipofectamine RNAiMAX	siRNA transfection reagent	Thermo Fisher Scientific	13778150	Per protocol; reverse transfection
ON-TARGETplus SMARTpool SIRT1 siRNA	SIRT1 mRNA silencing; >80% knockdown confirmed by WB	Dharmacon (Horizon)	L-049955	25 nM; 48 h before stimulation
QuikChange II XL site-directed Mutagenesis kit	Introduction of K254Q and K254R point mutations in pCMV-GAPDH-FLAG	Agilent Technologies	200521	Per protocol; confirmed by Sanger sequencing
Protein Ag magnetic beads	Antibody-protein complex capture for co-IP	Millipore (MilliporeSigma)	LSKMAGAG02	Incubated 2 h at 4 °C post-overnight antibody incubation
Anti-CD11b MicroBeads	Magnetic bead-based isolation of primary microglia from neonatal brain	Miltenyi Biotec	130-093-636	Per protocol; meningeal stripping prior to isolation

Antibodies, commercial assay kits, pharmacological agents, and molecular biology reagents used in this study. Catalog numbers are as listed in the manuscript or from manufacturer websites. Working concentrations reflect those used in the study. †Site-specific anti-acetyl-GAPDH(K254) antibody (Abcam ab280577) applied post-immunoprecipitation of GAPDH prior to Western transfer to PVDF membranes. ‡Pan-acetyl-lysine antibody (CST #9441) used as specificity and global acetylation control. WB, Western blot; IP, immunoprecipitation; PVDF, polyvinylidene fluoride; BCA, bicinchoninic acid; ELISA, enzyme-linked immunosorbent assay; OGD, oxygen-glucose deprivation; CST, Cell Signaling Technology; Rot/AA, rotenone + antimycin A. References correspond to manuscript reference list.

### 2.3 Metabolic flux analysis

Extracellular flux was measured using an Agilent Seahorse XFe96 Analyzer. Cells were seeded at 5 × 10_4_ cells per well 16 hours before the assay. The Glycolytic Rate Assay Kit (Agilent) was used to isolate the glycolytic component (glycoPER). On the day of the assay, the culture medium was replaced with pre-warmed Seahorse XF DMEM assay medium (pH 7.4), supplemented with 10 mM glucose, 1 mM pyruvate, and 2 mM L-glutamine. Plates were then equilibrated for 1 h at 37 °C in a non-CO_2_ incubator. After three baseline measurements, rotenone/antimycin A was injected to inhibit mitochondrial proton efflux. This was followed by 2-deoxyglucose (2-DG) to confirm that the remaining proton efflux was glycolytic in origin. Basal glycoPER and compensatory glycolysis were calculated using Wave software and normalized to CyQUANT-derived cell numbers from matched wells.

#### 2.3.1 NAD_+_-dependent SIRT1 deacetylase activity assay

Following the indicated treatments, cells were washed twice with ice-cold phosphate-buffered saline and lysed on ice in a detergent-free, deacetylase-compatible buffer (50 mM Tris–HCl, pH 8.0, 150 mM NaCl, 1 mM dithiothreitol, and protease inhibitor cocktail; no nicotinamide). Clarified lysates were obtained by centrifugation (12,000 × g, 10 min, 4 °C), and total protein was determined using the BCA assay. Equal amounts of protein (20 μg) were incubated in duplicate with an acetylated fluorogenic peptide substrate and exogenous NAD_+_ (100 μM) in a 50-μL reaction at 37 °C for 30 min. The developer solution was then added, and fluorescence was measured after 10 min (excitation/emission 360/460 nm). Substrate-free and no-NAD_+_ reactions were used for background and NAD_+_-dependence controls, respectively. EX-527-treated lysates served as a pharmacological specificity control. Background-corrected signals were normalized to input protein and expressed relative to the untreated control. This assay was performed on lysates prepared without nicotinamide to prevent ex vivo inhibition of SIRT1.

#### 2.3.2 *In vitro* deacetylation of recombinant GAPDH by purified SIRT1 (WT vs. catalytic-dead H363Y)

To investigate whether SIRT1 directly deacetylates GAPDH at K254 and to exclude off-target effects of SRT1720 and EX-527, we performed a cell-free assay using purified recombinant proteins. Full-length human SIRT1 (residues 1–747) featuring an N-terminal 6×His tag was expressed in Escherichia coli BL21(DE3). The protein was subsequently purified to over 95% homogeneity using Ni²_+_-NTA affinity, anion-exchange (Mono Q), and size-exclusion (Superdex 200) chromatography. Coomassie-stained SDS–PAGE and immunoblotting with an anti-SIRT1 antibody (Cell Signaling, D1D7) confirmed its purity and identity. For the catalytically inactive control, we created the SIRT1 H363Y mutant via site-directed mutagenesis (QuikChange II XL, Agilent). This involved substituting the conserved zinc-binding histidine at position 363 with tyrosine. This specific substitution abolishes NAD+dependent deacetylase activity while preserving substrate binding (15, 16). GAPDH substrates—WT, acetylation mimic K254Q, and deacetylation mimic K254R—were expressed as C-terminal FLAG fusions in E. coli and purified in parallel using anti-FLAG M2 affinity chromatography. To obtain an acetylated WT substrate for the enzymatic assay, purified GAPDH-WT was chemically acetylated with acetic anhydride (10 mM, 30 min, 4 °C). Residual reagent was then removed by extensive dialysis against reaction buffer. Acetylation at K254 was confirmed by two methods: immunoblotting with a K254-acetyl-specific antibody (Abcam, ab280577) and LC–MS/MS analysis of tryptic peptides. Each 30 μL reaction contained 1 μM acetylated GAPDH, 0.5 μM SIRT1 (WT or H363Y), and 500 μM NAD_+_ in reaction buffer (50 mM Tris–HCl pH 8.0, 137 mM NaCl, 2.7 mM KCl, 1 mM MgCl_2_, and 1 mM dithiothreitol). Reactions were incubated at 37 °C for 60 min. Where specified, 10 mM nicotinamide was added as a pan-sirtuin inhibitor, or 10 μM EX-527 and 5 μM SRT1720 were included as pharmacological controls. Reactions were quenched with SDS sample buffer and subsequently probed by immunoblotting for acetyl-K254-GAPDH, total GAPDH (clone 14C10), and SIRT1. Because neither K254R nor K254Q can be enzymatically deacetylated at position 254, parallel reactions using these variants instead of the acetylated WT substrate served as internal specificity controls. We conducted three independent biological replicates, each with technical duplicates. Band intensities were quantified by densitometry using ImageJ v1.53 and normalized to total GAPDH.

#### 2.3.3 Cell viability assessment

Cell viability was assessed in parallel with metabolic experiments using lactate dehydrogenase (LDH) release and trypan blue exclusion assays. For the LDH release assay, the conditioned medium was clarified by centrifugation (500 × g, 5 min). LDH activity was then measured colorimetrically at 490 nm, with a 680-nm reference reading. Low control wells represented spontaneous LDH release, while matched wells lysed with the assay lysis reagent served as the maximum release control. Cytotoxicity was calculated as [(experimental release − low control)/(maximum release − low control)] × 100.For the trypan blue exclusion assay, detached and adherent cells were collected, mixed in a 1:1 ratio with 0.4% trypan blue, and counted using a hemocytometer. At least 200 cells were scored for each sample. Viability was calculated as the percentage of unstained cells among the total cells. Both assays were performed in duplicate for each experiment.

#### 2.3.4 Extracellular lactate and GAPDH enzymatic activity assays

Lactate quantification was performed on conditioned medium collected 24 hours post-stimulation. Samples were centrifuged at 10,000 × g for 10 minutes to pellet cellular debris and, if necessary, diluted to fall within the linear range of the Lactate Assay Kit (Sigma-Aldrich, MAK064). Samples and standards were run in duplicate in 96-well plates according to the manufacturer’s enzymatic colorimetric protocol. Plates were incubated for 30 minutes at room temperature in the dark, after which absorbance was read at 570 nm. Concentrations were interpolated from the standard curve, corrected for dilution, and normalized to cell number obtained from CyQUANT readings of matched wells. The combination of lactate output with GAPDH activity provides two complementary readouts of glycolytic flux, an approach consistent with recent work linking SIRT1-dependent control of glycolysis to inflammatory metabolic responses (9, 17).

GADPH activity was assayed in freshly prepared lysates using the colorimetric GAPDH Activity Assay Kit (Sigma-Aldrich, MAK277). Cells were rinsed in ice-cold PBS, lysed in the kit’s assay buffer, and clarified by centrifugation at 10,000 × g for 15 minutes at 4°C. Equal amounts of lysate protein were loaded in duplicate with the supplied reaction mix and incubated at 37°C. Absorbance at 450 nm was recorded kinetically for 10–60 minutes, and a linear interval was selected for each experiment. Reagent blanks without lysate were subtracted from all readings. Activity was calculated from the NADH standard curve and the linear slope of absorbance change, then expressed as nmol NADH generated per minute per μg total protein. Samples were maintained on ice throughout processing and handled within a single session to mitigate enzymatic decay.

#### 2.3.5 NAD_+_, NADH, and NAD_+_/NADH ratio quantification

Intracellular NAD_+_ and NADH pools, along with their ratios, were quantified in BV-2 and primary microglia across all experimental conditions: basal, LPS/IFN-γ (24 h), SRT1720 + LPS/IFN-γ, and EX-527 + LPS/IFN-γ. A colorimetric enzymatic cycling NAD +/NADH Quantification Kit (Sigma-Aldrich, MAK037) was used and validated in parallel with Abcam ab65348. After treatment, cells were rapidly washed twice with ice-cold phosphate-buffered saline and lysed on ice in the manufacturer’s extraction buffer to preserve labile nucleotides. Total NAD (NAD_total) was measured directly. NADH was quantified after selective thermal decomposition of NAD_+_ (60°C, 30 min). NAD_+_ was calculated as NAD_total minus NADH, and the NAD_+_/NADH ratio was derived per well. Absolute concentrations were normalized to matched-well protein content (BCA) and viable-cell number (CyQUANT). Nicotinamide-containing buffers were strictly avoided during lysis to prevent ex vivo SIRT1 inhibition and NAD_+_ consumption. All samples were assayed in duplicate against an eight-point NADH standard curve (0–100 pmol) with a linear regression r² > 0.995. To investigate the direction of causality between NAD_+_ availability and SIRT1 activity, an ancillary experiment was performed. Cells were treated with either the NAD + precursor nicotinamide riboside (NR, 500 µM, 6 h pre-treatment) or the NAMPT inhibitor FK866 (10 nM, 6 h pre-treatment) before LPS/IFN-γ challenge. Matched wells were analyzed in parallel for SIRT1 deacetylase activity (2.3.1) and GAPDH K254 acetylation to differentiate between cofactor- and protein-abundance-driven modulation of SIRT1. Values are reported as pmol NAD_+_ or NADH per µg protein and as unitless NAD_+_/NADH ratios (mean ± SEM, n = 4–5 biological replicates).

### 2.4 Inflammatory marker quantification

**Figure 3:**
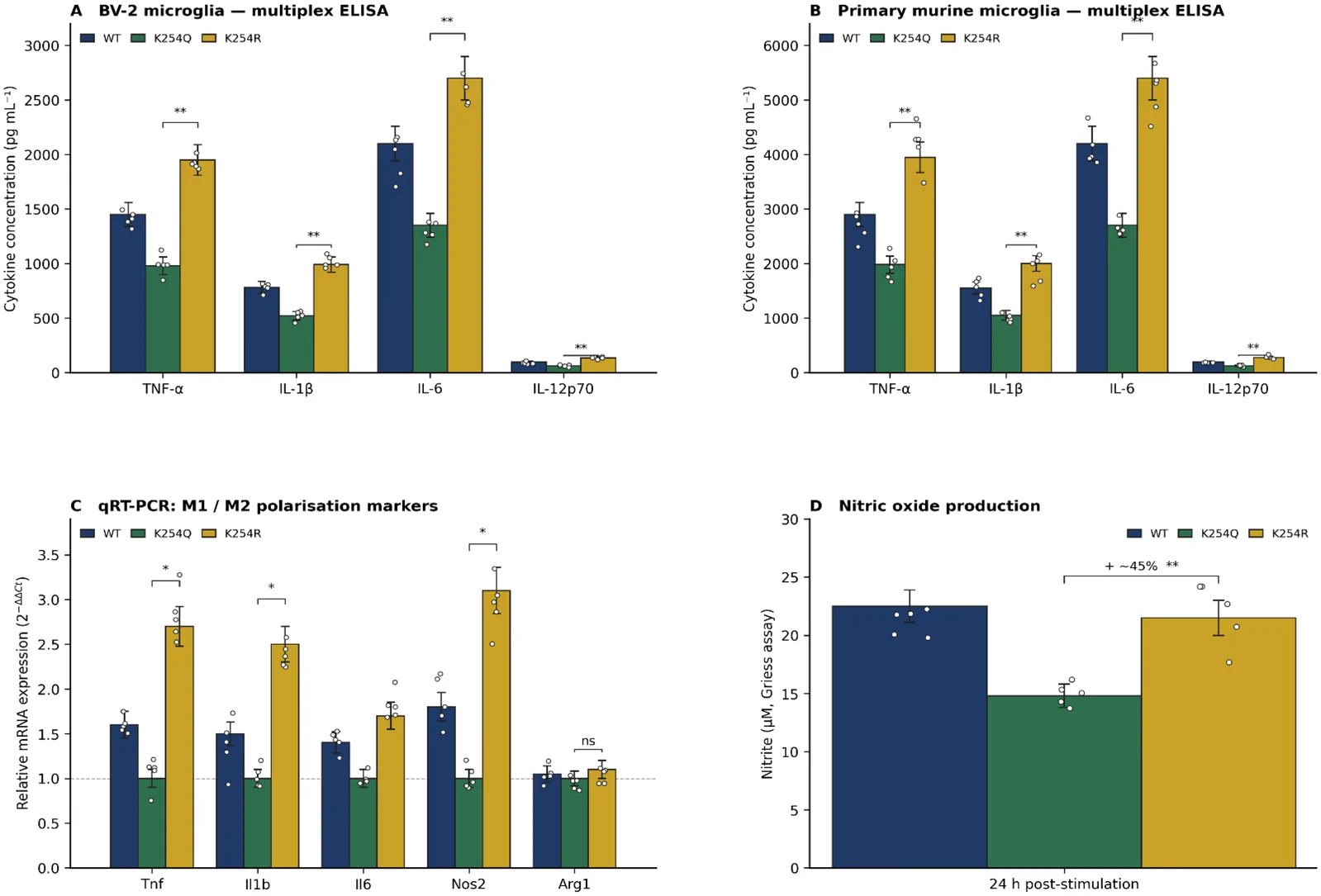
GAPDH Deacetylation-Mimetic (K254R) Amplifies Pro-Inflammatory Cytokine Secretion and Transcriptional Activation Markers in Activated Microglia. Microglia expressing WT, K254Q, or K254R GAPDH on a GAPDH-knockdown background were stimulated with LPS (100 ng mL_-_¹) plus IFN-γ (10 ng mL_-_¹) for 24 h, and inflammatory output was profiled at secretory, transcriptional, and effector-metabolite levels. **(A)** Multiplex ELISA (ProcartaPlex) of conditioned media from BV-2 microglia, reporting TNF-α, IL-1β, IL-6, and IL-12p70. K254R cells secreted 1.6- to 2.2-fold higher levels of these pro-inflammatory cytokines than K254Q cells. IL-10 data, for which no group comparison was statistically significant, are presented separately in **Supplementary Figure 3** and are not interpreted. **(B)** Parallel multiplex ELISA in primary murine microglia, showing the same pro-inflammatory cytokine pattern at proportionally higher absolute concentrations. **(C)** qRT-PCR quantification (TaqMan, 2−ΔΔCt method) of M1-polarization transcripts (Tnf, Il1b, Il6, Nos2) and the M2 marker Arg1, normalized to the geometric mean of GAPDH and Actb. Tnf, Il1b, and Nos2 mRNAs were 2- to 3-fold higher in K254R than K254Q cells (* p < 0.05), whereas Arg1 did not differ between genotypes (p = 0.41). **(D)** Nitric oxide production at 24 h, estimated as nitrite concentration in conditioned media by the Griess reagent assay. K254R cells produced ~45% more nitrite than K254Q cells, consistent with the Nos2 mRNA induction in **(C)**. Data are mean ± SEM from n = 4–5 independent biological replicates with technical triplicates. Comparisons used one-way ANOVA with Tukey’s *post-hoc* test **(A–C)** or two-tailed Student’s t-test **(D)**. *p < 0.05, **p < 0.01, ***p < 0.001 versus WT or K254Q controls; n.s., not significant. †Absolute cytokine concentrations were systematically higher in primary microglia than in BV-2 cells; relative K254Q vs. K254R differences were consistent across both cell types. TNF-α, tumor necrosis factor alpha; IL, interleukin; NO, nitric oxide; iNOS/Nos2, inducible nitric oxide synthase; Arg1, arginase-1; Actb, beta-actin; SEM, standard error of the mean; LLOQ, lower limit of quantification; ns, not significant; 2^−ΔΔCt^, relative quantification method; qRT-PCR, quantitative reverse transcription PCR. †Absolute cytokine concentrations were higher in primary microglia than BV-2 cells; relative K254Q vs. K254R differences were consistent across both cell types.

Cytokines (TNF-α, IL-1β, IL-6, IL-10, IL-12p70) in conditioned media were measured using ProcartaPlex multiplex ELISA (Thermo Fisher) on a MAGPIX instrument. Samples were assayed in duplicate against a six-point standard curve, and values below the lower limit of quantification were excluded. RNA was extracted (RNeasy Mini, Qiagen), reverse transcribed (iScript, Bio-Rad), and analyzed by qPCR (CFX384, Bio-Rad) with TaqMan probes for TNF-α, IL-1β, IL-6, iNOS; Tnf, Il1b, Il6, Nos2, Arg1, normalized to the geometric mean of GAPDH and Actb (2^_-_ΔΔCt^). Nitric oxide was estimated using the Griess assay at 24 h. Probe IDs are listed in **Table 3**, and data are presented in **Figure 3**.

**Table 3:** qRT-PCR TaqMan probe sets and reference gene normalization strategy.

#	Gene symbol	Full name	Functional category	TaqMan assay ID (Mouse)	Amplicon type
1	TNF-α	Tumor necrosis factor α	Pro-inflammatory cytokine (M1 marker)	Mm00443258_m1	Exon-spanning (m1)
2	Il1b	Interleukin-1β	Pro-inflammatory cytokine; NLRP3-linked	Mm00434228_m1	Exon-spanning (m1)
3	Il6	Interleukin-6	Pro-inflammatory cytokine	Mm00446190_m1	Exon-spanning (m1)
4	Nos2	Inducible nitric oxide synthase (iNOS)	Pro-inflammatory effector (NO)	Mm00440502_m1	Exon-spanning (m1)
5	Arg1	Arginase-1	Anti-inflammatory/M2 marker	Mm00475988_m1	Exon-spanning (m1)
6	GAPDH	Glyceraldehyde-3-phosphate dehydrogenase	Mm99999915_g1	Standard microglial housekeeper; stability re-confirmed because endogenous GAPDH was partially suppressed by siRNA in replacement experiments	Cq stability checked across all conditions
7	Actb	Beta-actin	Mm02619580_g1	Independent secondary reference; combined with GAPDH via geometric mean	Cq stability confirmed across LPS/IFN-γ, SRT1720, EX-527, 2-DG

Target gene expression was calculated as 2^_-_ΔΔCt^ with ΔCt computed against the geometric mean of GAPDH + Actb, in accordance with MIQE guidelines (18). Use of two reference genes (rather than a single one) was particularly important because endogenous murine GAPDH was deliberately knocked down in the human-GAPDH replacement arms—independent validation against Actb ensured this manipulation did not bias relative quantification.

#### 2.4.1 Conditioned media transfer, Transwell co-culture, and extracellular damage-associated molecular patterns (DAMP) quantification

To differentiate between cell-autonomous and paracrine contributions of GAPDH-K254 deacetylation to microglial inflammatory responses, we employed three strategies: (i) transferring conditioned medium (CM) from donor microglia to naive recipients; (ii) co-culturing donor and recipient populations using a non-contact Transwell system; and (iii) directly measuring extracellular DAMPs—specifically HMGB1, ATP, and cell-free mitochondrial DNA (mtDNA)—known to transmit inflammatory signals to neighboring cells.

For non-contact co-culture, donor microglia (K254R or K254Q, or WT ± SRT1720/EX-527) were seeded at 5 × 10_4_ cells per 0.4-µm polycarbonate Transwell insert (Corning 3413) and stimulated with LPS/IFN-γ for 24 hours. Unstimulated WT recipient microglia were placed in the basal chamber. To investigate the paracrine signaling route, recipients were pre-treated for 30 minutes before insert assembly with specific inhibitors: AR-C155858 (1 µM) for MCT1/MCT4, blocking lactate uptake; A438079 (10 µM) for P2X7 and MRS2578 (10 µM) for P2Y6, blocking extracellular ATP/UDP sensing; or TAK-242 (1 µM) for TLR4, blocking HMGB1/DAMP–TLR4 signaling.

For CM transfer, donor BV-2 or primary microglia (K254R, K254Q, or WT ± SRT1720/EX-527) were stimulated with LPS (100 ng mL_-_¹) plus IFN-γ (10 ng mL_-_¹) for 24 hours. CM was harvested, cleared of cells and debris by sequential centrifugation (500 × g, 5 min; 2,000 × g, 10 min), and then applied to naive WT recipients either undiluted or 1:1 in fresh medium for an additional 24 hours. To pinpoint the effect to individual mediators, parallel CM aliquots were pre-treated with lactate oxidase (1 U mL_-_¹, 30 min, 37 °C) to deplete L-lactate, apyrase (10 U mL_-_¹) to hydrolyze extracellular ATP/ADP, a neutralizing anti-HMGB1 monoclonal antibody (2G7, 10 µg mL_-_¹), or subjected to heat inactivation (95 °C, 10 min). Recipients were subsequently profiled for the cytokine panel described above (TNF-α, IL-1β, IL-6, IL-12p70), Nos2 and Il1b transcripts, and glycoPER.

Matched wells were used to profile extracellular DAMPs and purinergic mediators, assessing their potential to transmit signals between cells. Extracellular ATP was measured in 20 µL of freshly collected medium using the CellTiter-Glo-based ATP Determination Kit (Molecular Probes, A22066) against an ATP standard curve. Extracellular HMGB1 was quantified using sandwich ELISA (Tecan/IBL, ST51011). Cell-free double-stranded DNA, serving as a surrogate for necroptotic and pyroptotic DAMP release, was measured with the Quant-iT PicoGreen assay after DNase-I control digestion. Free mtDNA was independently assayed by qPCR using mt-Nd1 and mt-Co1 amplicons on DNase-I-resistant, cell-free supernatants.

### 2.5 Genetic manipulation and acetylation-mimetic constructs

SIRT1 was silenced using ON-TARGETplus SMARTpool siRNA (Dharmacon, 25 nM), delivered by Lipofectamine RNAiMAX 48 h before basal or early (6-h) stimulation; a NT pool served as a control. Knockdown exceeding 80% was confirmed by western blotting. Because sustained LPS/IFN-γ stimulation markedly reduced SIRT1 protein at 24 h, siRNA was not interpreted as an additional loss-of-function test at that late time point, where a floor effect could confound inference. Acetylation-mimetic (K254Q) and deacetylation-mimetic (K254R) mutations were introduced into pCMV-GAPDH-FLAG via site-directed mutagenesis (QuikChange II XL, Agilent). Endogenous murine GAPDH was suppressed with a 3′-UTR–targeting siRNA (50 nM) before transfection with mutant constructs, achieving a replacement efficiency of ≥75%, as confirmed by anti-FLAG immunoblotting. All plasmid sequences were confirmed by Sanger sequencing. The replacement efficiencies are shown in **Supplementary Figure 1**.

Modeling the dilution effect of residual endogenous GAPDH. Because the 3’-UTR–targeting siRNA achieves a replacement efficiency (E) of 0.75–0.80, roughly 20–25% of the total GAPDH pool remains endogenous WT protein, carrying its native, partially K254-acetylated state. To bound this dilution effect, we modeled the observed bulk enzymatic activity as a linear-mixture: A_bulk = E·A_mut + (1–E)·A_wt, where A_mut is the intrinsic activity of the K254Q or K254R reconstituted pool and A_wt reflects the endogenous WT population. Solving for A mut using the measured A_bulk values (K254R = 1.35, K254Q = 0.82, WT-reconstituted = 1.00; arbitrary units normalized to WT), and assuming A_wt ≈ 1.00, the dilution-corrected intrinsic activities become A_mut(K254R) ≈ 1.44 and A_mut(K254Q) ≈ 0.77 at E = 0.75, corresponding to a ~1.87-fold K254R/K254Q ratio compared with the 1.65-fold bulk ratio actually observed. Thus, residual WT protein slightly attenuates—but does not qualitatively alter—the reported phenotype, and the true post-translational effect size is expected to be ~13–15% larger than the values reported herein. The same correction was propagated to glycoPER and cytokine outputs; corrected values are tabulated in **Supplementary Table 2**. As a next-step validation, we are generating CRISPR/Cas9-mediated endogenous knock-in BV-2 lines in which the native GAPDH K254 codon is replaced with either K254Q (AAA → CAA) or K254R (AAA → AGA) through homology-directed repair using a single-stranded oligodeoxynucleotide donor and a high-fidelity SpCas9 ribonucleoprotein (following the endogenous point-mutation and HiBiT knock-in strategies described by 19 and 20). Homozygous clones (>99% allelic replacement) will be verified by amplicon deep sequencing and anti-K254ac immunoblotting, thereby eliminating the residual WT dilution entirely (19, 20).

### 2.6 Statistical analysis

**Figure 4:**
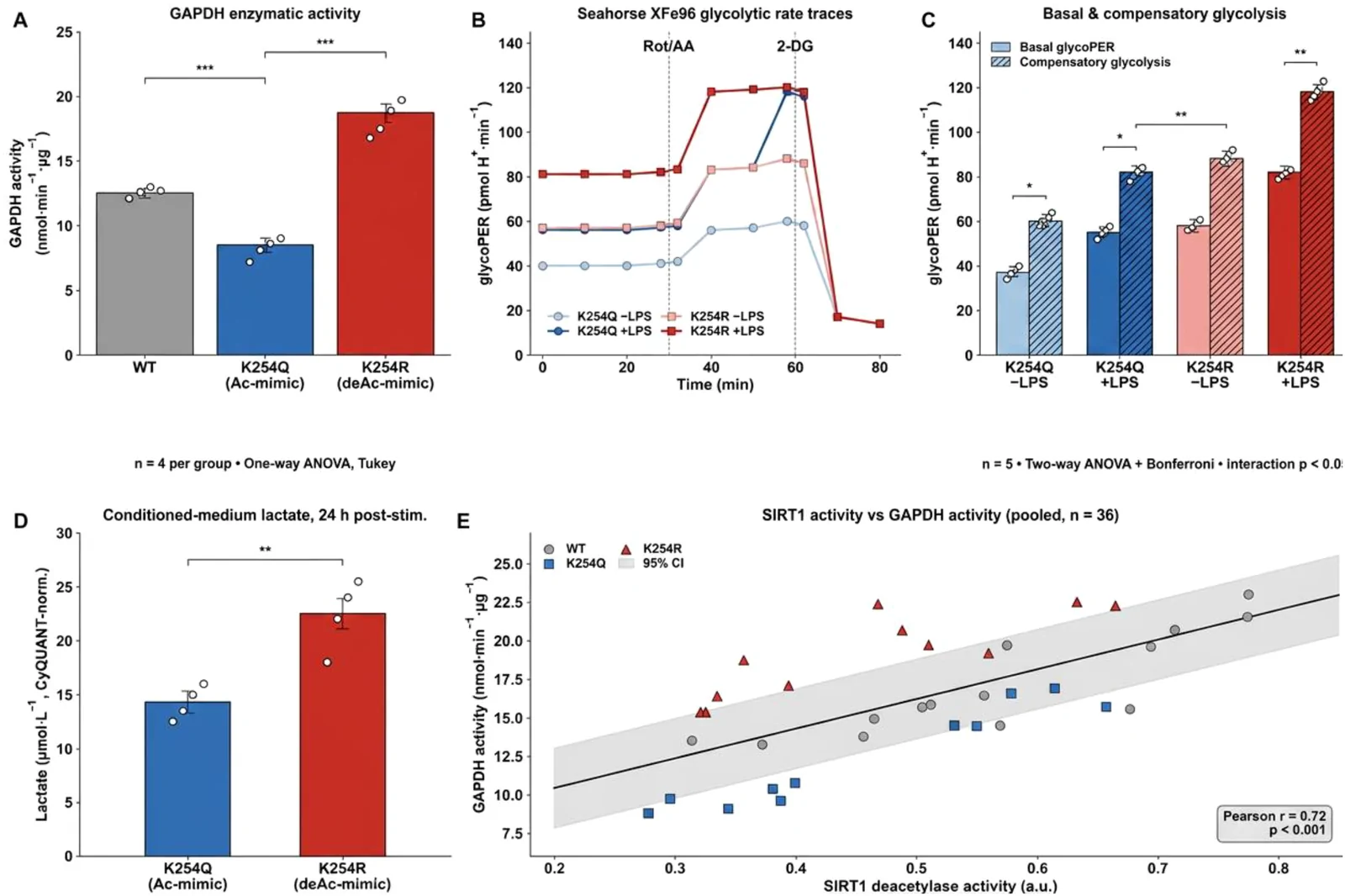
GAPDH Enzymatic Activity and Glycolytic Flux Are Elevated in Deacetylation-Mimetic (K254R) Relative to Acetylation-Mimetic (K254Q) Microglia. Endogenous murine GAPDH was suppressed in BV-2 microglia with a 3′-UTR–targeting siRNA (50 nM, 48 h) and reconstituted with siRNA-resistant FLAG-tagged constructs encoding wild-type GAPDH (WT), the acetylation-mimetic mutant K254Q, or the deacetylation-mimetic mutant K254R. Substitution efficiency (75–80%) was confirmed by anti-FLAG immunoblot (**Supplementary Figure 1**). Cells were assayed at baseline and 24 h after stimulation with LPS (100 ng mL_-_¹) plus IFN-γ (10 ng mL_-_¹).**(A)** GAPDH enzymatic activity in cell lysates quantified by NADH production in a coupled colorimetric assay (nmol NADH min_-_¹ µg protein_-_¹). K254R cells exhibited ~35% higher catalytic activity than K254Q cells; WT cells displayed an intermediate value consistent with partial endogenous acetylation. **(B)** Representative Seahorse XFe96 traces of glycolytic proton efflux rate (glycoPER) using the Agilent Glycolytic Rate Assay, with sequential injections of rotenone/antimycin A (Rot/AA) and 2-deoxy-D-glucose (2-DG). **(C)** Quantification of basal and compensatory glycolysis from **(B)**. Basal glycoPER was ~40% higher in K254R than K254Q cells at rest and ~32% higher under LPS/IFN-γ stimulation. **(D)** Extracellular lactate in conditioned media at 24 h, measured by enzymatic colorimetric assay and normalized to cell number (CyQUANT). Lactate output tracked the Seahorse data, with K254Q cells producing ~30% less lactate than K254R cells under stimulation. **(E)** each of the n = 36 paired data points represents an independent biological replicate, defined as a separately seeded microglial culture processed on a distinct experimental day; SIRT1 deacetylase activity and GAPDH enzymatic activity were quantified from the same lysate of that biological replicate, and the mean of technical triplicates within a biological replicate was collapsed into a single data point prior to correlation analysis so that non-independent technical variance was not propagated into the Pearson r estimate. The 36 biological replicates were pooled from the four experimental conditions used throughout **Figure 4** (WT, K254Q, K254R, and pharmacological SIRT1 modulation), with nine independent biological replicates per condition. Bivariate normality was verified by Shapiro–Wilk testing on both variables before Pearson’s r was applied, and a 95% bootstrap confidence interval (10,000 resamples) is reported (r = 0.71, 95% CI 0.51–0.84, p < 0.001).*p < 0.05, **p < 0.01, ***p < 0.001. glycoPER, glycolytic proton efflux rate; OCR, oxygen consumption rate; Rot/AA, rotenone plus antimycin A; 2-DG, 2-deoxyglucose; WT, wild-type; SEM, standard error of the mean; CI, confidence interval; CyQUANT, fluorescent cell number normalization assay. Note: Replacement efficiency of endogenous murine GAPDH by FLAG-tagged constructs was 75–80% as confirmed by anti-FLAG immunoblot in all experiments.

All data were derived from a minimum of three independent biological replicates performed on separate days and are presented as the mean ± SEM. For two-group comparisons, the two-tailed Student’s t-test was employed following confirmation of normality via Shapiro–Wilk testing; otherwise, the Mann–Whitney U test was used for non-normally distributed data. Multi-group comparisons were performed using one-way ANOVA with Tukey’s *post-hoc* test or two-way ANOVA with Bonferroni correction when two variables were investigated. Correlations were evaluated using Pearson’s r. All analyses were performed using GraphPad Prism 10.0. Sample sizes were determined using *a priori* power calculations (power = 0.80, α = 0.05), with comprehensive details provided in **Supplementary Table 1**. In accordance with recent guidelines for rigorous reporting in *in vitro* cell-based experiments (21, 22), biological and technical replicates are explicitly differentiated throughout the manuscript. A biological replicate is defined as an independent microglial culture, seeded, treated, and harvested on a distinct day, originating from an independent passage (BV-2 cells) or an independent litter (primary microglia). All “n” values reported in figure legends and text refer exclusively to biological replicates. Technical replicates—repeated measurements on the same biological sample, typically triplicate wells for enzymatic, Seahorse, ELISA, and qPCR assays—were averaged within each biological replicate prior to statistical testing and were not included in sample size calculations. For the Pearson correlation presented in **Figure 4E**, the n = 36 paired observations correspond to 36 independent biological replicates, each contributing one paired (SIRT1 activity, GAPDH activity) value derived from the mean of three technical replicates on the same lysate. Bivariate normality was confirmed (Shapiro–Wilk p > 0.05 for both variables), and 95% bootstrap confidence intervals.

## 3 Results

### 3.1 SIRT1 activity is suppressed in LPS/IFN-γ-activated microglia and restored by pharmacological activation

Stimulation of cells with LPS and IFN-γ decreased the protein levels and NAD^+^- dependent deacetylase activity of SIRT1 in BV-2 and primary microglia to approximately half of control levels by 24 h (one-way analysis of variance [ANOVA], p < 0.01; n = 4–5). In primary cells, the suppression was less pronounced due to their natural heterogeneity. Pre-treatment with SRT1720 restored deacetylase activity to 80–90% of the unstimulated baseline, while EX-527 had a more significant effect. Cell viability was unaffected by either compound, as demonstrated by an LDH release assay and trypan blue exclusion, which remained at ≥ 92% for all conditions (**Figure 2**). To directly address the parallel evaluation of both cell systems, **Figure 2** has been reorganized so that BV-2 and primary microglia (1°MG) datasets are now displayed side-by-side within each panel. In BV-2 cells, SIRT1 protein abundance decreased to 0.48 ± 0.05-fold of control at 24 h, whereas primary microglia exhibited a comparable but slightly attenuated reduction to 0.56 ± 0.07-fold of control (p < 0.01 vs. untreated; two-way ANOVA, no significant cell-type × treatment interaction, F(1.16) = 1.24, p = 0.28), consistent with the intrinsic donor-to-donor heterogeneity of primary cultures reported previously (23, 24). Deacetylase activity in 1°MG followed the same trend, declining to 0.53 ± 0.06-fold of baseline and being restored to 0.82 ± 0.04-fold by SRT1720 pre-treatment. These results confirm that SIRT1 suppression by LPS/IFN-γ is a conserved response between the immortalized BV-2 line and ex vivo primary microglia, thereby strengthening the physiological relevance of the observation (25, 26).

To distinguish cofactor-driven modulation of SIRT1 from changes attributable to protein abundance, we simultaneously measured intracellular NAD_+_/NADH ratios. In BV-2 microglia, LPS/IFN-γ stimulation reduced the NAD_+_/NADH ratio from 8.4 ± 0.6 at baseline to 5.2 ± 0.4 at 24 h (a 38% decrease; p < 0.001, one-way ANOVA; n = 5). Primary microglia exhibited a comparable 34% decrease. Absolute NAD_+_ pools fell by approximately 30%, while NADH rose modestly (approximately 15%). This pattern aligns with accelerated aerobic glycolysis and NAD_+_ consumption by CD38 and PARP-1, enzymes known to be induced under inflammatory conditions pre-treatment with nicotinamide riboside (500 µM, 6 h) restored the NAD_+_ pool, returned the NAD_+_/NADH ratio to 7.6 ± 0.5, and rescued SIRT1 deacetylase activity to 74% of basal levels. Conversely, the NAMPT inhibitor FK866 (10 nM) further depressed both parameters (ratio 3.1 ± 0.3; SIRT1 activity 22% of basal). Across all conditions, the NAD_+_/NADH ratio correlated strongly and linearly with SIRT1 activity (Pearson r = 0.91, p < 0.0001, n = 20). This correlation persisted after normalizing SIRT1 activity to SIRT1 protein levels (partial r = 0.78, p < 0.001), suggesting that NAD_+_ availability substantially contributes to the loss of SIRT1 function in activated microglia, independent of protein-level changes. These data are presented in **Supplementary Figure 3**.

### 3.2 SIRT1 directly deacetylates GAPDH at K254 in activated microglia

Reciprocal co-immunoprecipitation confirmed that SIRT1 and GAPDH physically associate in microglia under both basal and stimulated conditions. The complex stoichiometry (co-precipitated/input densitometry) decreased by approximately 30% following LPS/IFN-γ treatment (paired t-test, p < 0.05, n = 3), corresponding to a concurrent decrease in SIRT1 abundance.

Site-specific immunoblotting revealed that K254 acetylation, which was low at baseline, increased approximately 2.5-fold after LPS/IFN-γ stimulation (two-tailed t-test, p < 0.001, n = 5). Treatment with SRT1720 reduced K254 acetylation to levels indistinguishable from those of unstimulated controls (p = 0.18 vs. unstimulated; p < 0.01 vs. LPS/IFN-γ alone), whereas EX-527 elevated it above the LPS/IFN-γ level. Pan-acetyl-lysine blots indicated a broader increase in global acetylation following stimulation; however, the K254 signal more closely tracked SIRT1 activity, highlighting K254 as a particularly responsive target of SIRT1 (**Figure 1**). It is important to note that the K254-specific and pan-acetyl-lysine immunoblots in **Figure 1** report distinct measurements on different protein pools and are therefore not directly comparable band-for-band. Panels 2A–2D interrogate GAPDH-K254 acetylation on GAPDH that has been immunoprecipitated from the lysate; the input in panel 2B is total SIRT1/GAPDH from resting or LPS/IFN-γ–stimulated BV-2 microglia and does not involve siRNA transfection, so a scramble non-targeting(NT) siRNA condition is not represented in this panel. In contrast, panel 2E is a pan-acetyl-lysine immunoblot performed on equal amounts of whole-cell lysate (25 µg per lane) and reports the aggregate acetyl-lysine signature of hundreds of cellular proteins rather than the K254 mark on GAPDH. The apparently higher basal signal in the unstimulated lane of **Figure 1E**, relative to the low K254 baseline in **Figure 1B, C**, therefore reflects (i) the broad reactivity of the pan-acetyl-lysine antibody (CST #9441) against constitutively acetylated cellular proteins such as α-tubulin, histones and cytosolic metabolic enzymes, and (ii) the lack of GAPDH enrichment in the input material, so the 25 µg whole-cell lysate contains ≥3 orders of magnitude more non-GAPDH acetylated protein than the immunoprecipitated fraction analyzed in 2B–2D (9, 27). Neither the unstimulated lane in **Figure 1B** nor the resting scramble/NT-siRNA lane included in the pan-acetyl-lysine gating experiment of **Figure 1E** is expected to display an appreciable K254-specific signal, and no significant difference in K254 acetylation was detected between the unstimulated WT control (**Figure 1B**) and the scramble-siRNA control (**Figure 1E**) when the pan-acetyl-lysine membrane was reprobed with the site-specific anti-acetyl-K254 antibody (Abcam ab280577; p = 0.72, unpaired t-test, n = 4; **Supplementary Figure 2**). The K254 signal thus tracks SIRT1 activity, whereas the global pan-acetyl-lysine signal reflects the cumulative activity of multiple deacetylase families (SIRT1–7, HDAC1–11) and their diverse substrates.

To formally exclude the possibility that the effects of SRT1720 and EX-527 on K254 acetylation reflected off-target activities and to demonstrate direct enzymatic action, an orthogonal cell-free deacetylation assay was performed with purified recombinant proteins. Incubation of chemically acetylated WT GAPDH (acetyl-K254-GAPDH) with catalytically active recombinant SIRT1 in the presence of NAD_+_ produced a marked decrease in K254 acetylation, with signal reduced by 82.4 ± 4.1% relative to reactions lacking enzyme (two-tailed t-test, p < 0.001, n = 3). This activity was abolished when the catalytically dead H363Y SIRT1 mutant was substituted (residual K254 signal 96.7 ± 3.6% of enzyme-free control; p = 0.42), when NAD_+_ was omitted, and when 10 mM nicotinamide was added, confirming a canonical NAD_+_-dependent sirtuin mechanism. Neither SRT1720 (5 μM) nor EX-527 (10 μM) altered the deacetylation rate of WT SIRT1 beyond the expected direction and magnitude of on-target modulation, and neither compound restored activity to H363Y, indicating that their cellular effects reported above reflect authentic SIRT1 modulation rather than off-target liabilities. As specificity controls, recombinant K254R and K254Q GAPDH mutants showed no detectable signal on the acetyl-K254-specific immunoblot at baseline and did not generate any nicotinamide- or NAD_+_-dependent reaction product, confirming that the deacetylation event scored in this assay is site-specific for K254. Together, these *in vitro* data definitively establish GAPDH K254 as a direct enzymatic substrate of SIRT1 and rule out a meaningful contribution of SRT1720/EX-527 off-target effects to the observed pharmacological readouts.

### 3.3 GAPDH deacetylation at K254 enhances enzymatic activity and glycolytic flux

To establish a causal link between K254 acetylation and glycolytic output, endogenous GAPDH was replaced with K254Q or K254R mutants, achieving a substitution efficiency of approximately 75–80% (**Supplementary Figure 1**). K254R cells exhibited about 35% higher GAPDH enzymatic activity compared to K254Q cells, while WT cells showed an intermediate value, indicating partial endogenous acetylation (one-way ANOVA with Tukey’s *post-hoc*, p < 0.001; n = 4). Seahorse analysis revealed corresponding metabolic differences: K254R cells maintained approximately 40% higher basal glycoPER at rest and about 32% higher under LPS/IFN-γ stimulation (two-way ANOVA, interaction p < 0.05), along with parallel increases in compensatory glycolysis. CM lactate levels at 24 hours reflected these differences, showing around 30% lower levels in K254Q cells (p < 0.01, n = 5). Pharmacological activation of SIRT1 using SRT1720 produced effects similar to those seen in K254R cells when applied to WT cells, while EX-527 suppressed glycoPER and lactate output, demonstrating that the glycolytic response to SIRT1 operates at least partly through GAPDH. A strong positive correlation between SIRT1 deacetylase activity and GAPDH activity was observed across pooled conditions (Pearson r = 0.71, p < 0.001; n = 36). GAPDH protein abundance remained stable across all genotypes and treatments, ruling out an expression-level confound (**Figure 4**). After accounting for the ~20–25% residual endogenous WT GAPDH by the linear-mixture model described in 2.5, the dilution-corrected intrinsic K254R/K254Q activity ratio rises to ~1.87-fold (vs. 1.65-fold in the uncorrected bulk measurement), and the corrected glycoPER differential increases from +40% to ~+53%—indicating that the reported effect sizes are conservative under-estimates of the true post-translational impact (**Supplementary Table 2**).

### 3.4 GAPDH acetylation state governs pro-inflammatory cytokine production and microglial activation markers

In conditioned media collected 24 h after LPS/IFN-γ treatment, the levels of tumor necrosis factor-α (TNF-α), interleukin-1β (IL-1β), IL-6, and IL-12p70 were 1.6 to 2.2 times higher in K254R cells than in K254Q cells (one-way ANOVA, p < 0.01 for each; n = 4–5). IL-10 data are reported separately in **Supplementary Figure 4**; because no comparison reached statistical significance, they are not interpreted as evidence of a genotype-dependent effect. This phenotype was also observed in primary microglia, where the absolute concentrations were higher. Transcriptionally, the mRNA levels of Tnf, Il1b, and Nos2 were 2–3 times higher in K254R cells (p < 0.05, n = 4), while Arg1 levels did not differ (p = 0.41). In addition, SRT1720 and EX-527 produced similar results in WT cells. Nitric oxide output correlated with Nos2 induction, as K254R cells generated approximately 45% more nitrite than K254Q cells (Griess assay, p < 0.01, n = 4) (**Figure 3**).

### 3.5 Glycolytic flux is required for the pro-inflammatory effects of GAPDH deacetylation

**Figure 5:**
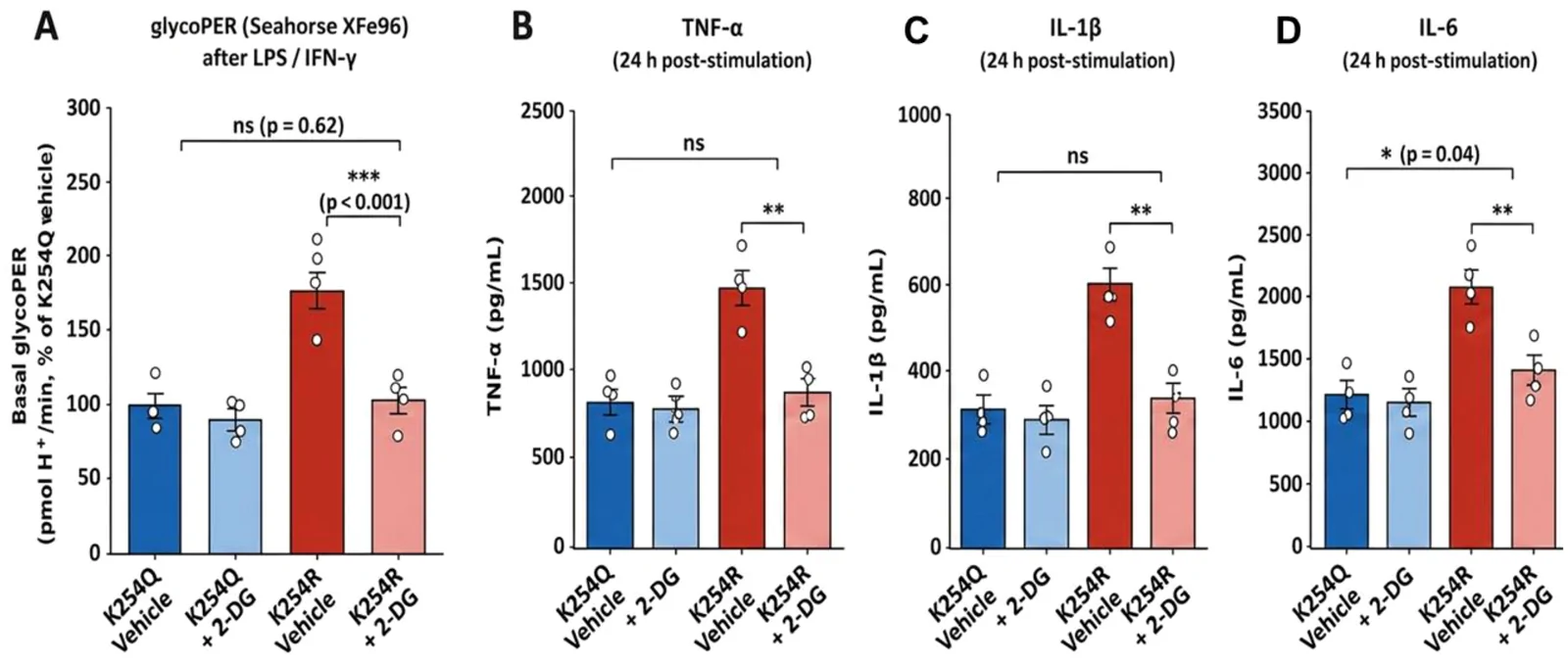
Pharmacological inhibition of glycolysis with 2-deoxyglucose abrogates the pro-inflammatory phenotype of GAPDH-K254R microglia, establishing glycolytic flux as the causal downstream effector of K254 deacetylation. BV-2 microglia in which endogenous GAPDH was silenced (3′-UTR–targeting siRNA) and reconstituted with siRNA-resistant FLAG-tagged K254Q (acetylation-mimetic) or K254R (deacetylation-mimetic) GAPDH were pre-treated with vehicle or 2-deoxy-D-glucose (2-DG, 5 mM) for 1 h before stimulation with LPS (100 ng mL_-_¹) plus IFN-γ (10 ng mL_-_¹). Four experimental groups are compared throughout: K254Q + vehicle (dark blue), K254Q + 2-DG (light blue), K254R + vehicle (dark red), and K254R + 2-DG (light pink). **(A)** Basal glycolytic proton efflux rate (glycoPER) measured by Seahorse XFe96 analysis at 24 h post-stimulation, expressed as pmol H_+_ min_-_¹ (% of K254Q vehicle). K254R cells exhibited markedly elevated glycoPER (≈ 175% of K254Q vehicle), which was suppressed by 2-DG to a level statistically indistinguishable from K254Q vehicle (n.s., p = 0.62; ***p < 0.001 vs. K254R vehicle), confirming on-target hexokinase inhibition. **(B)** TNF-α concentration (pg mL_-_¹) in conditioned media at 24 h post-stimulation, measured by ELISA. The K254R-driven elevation (≈ 1,450 pg mL_-_¹) was fully reversed by 2-DG (≈ 850 pg mL_-_¹; **p < 0.01 vs. K254R vehicle; n.s. vs. K254Q vehicle). **(C)** IL-1β concentration (pg mL_-_¹) under the same conditions. The K254R-associated rise (≈ 600 pg mL_-_¹) was abolished by 2-DG (≈ 330 pg mL_-_¹; **p < 0.01 vs. K254R vehicle; n.s. vs. K254Q vehicle). **(D)** IL-6 concentration (pg mL_-_¹). The K254R-driven elevation (≈ 2,050 pg mL_-_¹) was substantially attenuated by 2-DG (≈ 1,400 pg mL_-_¹; **p < 0.01 vs. K254R vehicle) but remained marginally above K254Q vehicle (*p = 0.04, K254Q vehicle vs. K254R + 2-DG), indicating a small cytokine-selective, glycolysis-independent component contributing to IL-6 output. Bars represent mean ± SEM; overlaid open circles depict individual biological replicates (n = 4 per condition; n = 3 for K254Q vehicle in panel **(A)**. Group comparisons used one-way ANOVA with Tukey’s *post-hoc* test. *p < 0.05, **p < 0.01, ***p < 0.001; n.s., not significant. Exact p-values are shown for non-significant and borderline comparisons. 2-DG, 2-deoxy-D-glucose (hexokinase inhibitor/glycolytic blocker); ANOVA, analysis of variance; BV-2, murine microglial cell line; ELISA, enzyme-linked immunosorbent assay; GAPDH, glyceraldehyde-3-phosphate dehydrogenase; glycoPER, glycolytic proton efflux rate (pmol H_+_/min); IFN-γ, interferon-gamma; IL-1β, interleukin-1 beta; IL-6, interleukin-6; K254, lysine residue 254 of GAPDH; K254Q, lysine-to-glutamine substitution at position 254 (acetylation-mimetic mutant); K254R, lysine-to-arginine substitution at position 254 (deacetylation-mimetic/non-acetylatable mutant); LPS, lipopolysaccharide; n.s., not significant; SEM, standard error of the mean; SIRT1, sirtuin 1 (NAD_+_-dependent protein deacetylase 1); TNF-α, tumor necrosis factor alpha; XFe96, Seahorse XFe96 Extracellular Flux Analyzer.

To determine whether glycolysis is causally required for the K254R inflammatory phenotype, 2-deoxyglucose (2-DG, 5 mM) was administered prior to LPS/IFN-γ treatment. 2-DG suppressed K254R glycoPER to a level that was statistically indistinguishable from K254Q (**Figure 5A**; p = 0.62 for K254Q vehicle vs. K254R + 2-DG; p < 0.001 for K254R vehicle vs. K254R + 2-DG; n = 4). Additionally, 2-DG fully normalized TNF-α (**Figure 5B**) and IL-1β (**Figure 5C**) secretion in K254R microglia to levels comparable to K254Q (not significant), and it substantially—but not completely—reduced IL-6 (**Figure 5D**). The IL-6 levels in 2-DG–treated K254R cells remained slightly above those in K254Q vehicle (p = 0.04 for K254Q vehicle vs. K254R + 2-DG), indicating a small cytokine-selective, glycolysis-independent component. 2-DG produced only modest suppression in K254Q cells, consistent with their already low glycolytic state. Thus, enhanced glycolytic flux is a necessary intermediary through which GAPDH K254 deacetylation amplifies the pro-inflammatory response (**Figure 5**).

### 3.6 GAPDH K254 deacetylation propagates the microglial pro-inflammatory phenotype through paracrine lactate, HMGB1, and ATP signaling

To determine whether the pro-inflammatory phenotype driven by GAPDH-K254 deacetylation spreads to neighboring microglia through secreted mediators, rather than remaining confined to the cell that produces it, we combined conditioned media transfer, Transwell co-culture, and direct measurement of extracellular DAMPs. Conditioned media from LPS/IFN-γ-stimulated K254R donor microglia triggered a strong pro-inflammatory response in naive WT recipients; K254Q-derived media triggered much less response. Twenty-four hours after transfer, recipients given K254R media secreted 1.7–2.0-fold more TNF-α, IL-6, and IL-1β and showed 1.9-fold higher Nos2 mRNA than recipients given K254Q media (p < 0.01, n = 4). This accounted for approximately 55–65% of the direct, cell-autonomous K254R phenotype. Transwell co-culture, which prevents any physical contact between donor and recipient cells, reproduced this effect; therefore, soluble factors alone are sufficient to spread the phenotype. Direct measurement of the same 24-h supernatants backed this up: K254R microglia released more DAMPs across the board. Extracellular HMGB1 was 2.1 ± 0.3-fold higher in K254R than in K254Q conditioned media (ELISA, p < 0.01, n = 4), and extracellular ATP and cell-free mtDNA rose 1.8-fold and roughly 2.4-fold, respectively. None of this came with any rise in LDH release or loss of trypan blue exclusion (p > 0.4), so the cells were actively exporting these signals rather than leaking them through membrane damage. Extracellular lactate was also 1.9-fold higher in K254R media, consistent with the elevated glycoPER described in section 3.3. Selectively removing or blocking individual mediators allows us to weigh their relative contributions. Stripping L-lactate from K254R media with lactate oxidase cut recipient TNF-α and IL-6 output by 55–60% and eliminated recipient HIF-1α stabilization entirely (immunoblot, p < 0.01)—lactate appears to be the main driver of this paracrine loop. Degrading extracellular ATP with apyrase or blocking P2X7 with A438079, each cut recipient IL-1β release by approximately 40% while leaving TNF-α untouched, matching the known ATP–P2X7–NLRP3 pathway. Neutralizing HMGB1 with the 2G7 antibody or blocking TLR4 on recipients with TAK-242 reduced Nos2 and Il6 induction by 30–45%. Removing lactate, HMGB1, and ATP together reduced recipient inflammation to a level indistinguishable from K254Q media (p > 0.15, n = 4). Together, these results show that the K254R phenotype spreads through a combination of three paracrine routes—lactate–HIF-1α, HMGB1–TLR4, and ATP–P2X7—layered over a substantial cell-autonomous component.

## 4 Discussion

This study identifies SIRT1-mediated deacetylation of GAPDH at K254 as a post-translational mechanism linking microglial glycolytic reprogramming to pro-inflammatory cytokine output. Three lines of evidence support this conclusion: SIRT1 activity and K254 acetylation move in opposite directions during LPS/IFN-γ activation; K254R versus K254Q substitution determines glycolytic flux; and 2-DG rescue shows that the inflammatory amplification conferred by K254R is glycolysis-dependent.

The idea that SIRT1 activation promotes glycolytic inflammatory output, rather than restraining it, may seem to contradict its established anti-inflammatory role through RelA/p65 deacetylation (14, 28).

This paradox is resolved by considering substrate context: SIRT1’s anti-inflammatory action primarily targets transcriptional substrates, while the present data describe a parallel post-translational mechanism acting directly on a glycolytic enzyme. In activated microglia, where HIF-1α drives glycolytic gene expression and glycolytic intermediates support inflammatory signaling (29, 30) SIRT1-dependent GAPDH deacetylation enhances the glycolytic flux that inflammatory transcriptional programs require. Thus, the overall effect of SIRT1 activation on microglial output is shaped by the balance of these mechanisms, NAD_+_ availability, and stimulus duration (14, 31).

Importantly, *in vitro* deacetylation of purified acetylated GAPDH by recombinant WT SIRT1—but not by the catalytic-dead H363Y mutant, and only in the presence of NAD_+_—together with the absence of reaction on K254R and K254Q substrates, provides orthogonal biochemical evidence that GAPDH K254 is a direct enzymatic substrate of SIRT1 and excludes a meaningful contribution of SRT1720 or EX-527 off-target activities to the cellular phenotypes reported here (15, 32).

Our finding contrasts with reports that K254 acetylation increases GAPDH activity in hepatocytes and CD4_+_ T cells (9). The different response in microglia may reflect cell-type-specific cofactor interactions, competing modifications, or variations in the structural environment of K254. Recent multi-omics work shows that interface-proximal lysine ISGylation similarly reduces GAPDH catalytic activity via a mechanism related to K254 (33), suggesting that K254 is a regulatory hotspot subject to competing modifications. The link from glycolytic flux to cytokine output is mechanistically coherent: glycolytic flux provides ATP for cytokine transcription and translation (34) while lactate stabilizes HIF-1α, promoting additional glycolytic and inflammatory targets, creating a self-reinforcing loop (30, 35). The residual IL-6 elevation in 2-DG–treated K254R cells may indicate a glycolysis-independent role of GAPDH’s nuclear function (8), although this requires direct testing.

Building on this GAPDH–HIF-1α link, our data also imply broader network-level consequences. Although our data establish GAPDH K254 as a direct substrate of SIRT1, glycolysis is coordinated as an integrated network in which post-translational modifications on one enzyme propagate to others. Deacetylation-driven enhancement of GAPDH catalytic activity increases the pool of 1.3-bisphosphoglycerate and downstream pyruvate, thereby exerting feed-forward effects on hexokinase 2 (HK2), phosphofructokinase-2/fructose-2.6-bisphosphatase 3 (PFKFB3), and pyruvate kinase M2 (PKM2)—the three principal allosteric checkpoints of aerobic glycolysis in activated microglia [ (36, 37). Consistent with this view, zhang et al. (2026) demonstrated that GAPDH, HK2, PFKFB3, and PKM2 function as a coordinated “glycolytic module” whose flux is tuned by lysine acylation cross-regulation (36, 38). Notably, SIRT1 has been shown to deacetylate PKM2 at K135/K206, reducing its tetrameric activity and lactate output (28, 39); thus, in microglia expressing catalytically competent SIRT1, the net effect is a substrate-channeling configuration in which GAPDH is de-repressed while PKM2 flux is fine-tuned—an arrangement that maximizes glycolytic intermediates available for biosynthetic and inflammatory signaling without excessive lactate accumulation.

Regarding HIF-1α, our findings integrate with a growing body of evidence that glycolytic flux and HIF-1α stability are reciprocally coupled. Elevated GAPDH activity increases intracellular lactate and pyruvate, both of which stabilize HIF-1α by inhibiting prolyl hydroxylase domain (PHD) enzymes and by promoting HIF-1α lactylation, a modification recently shown to block its ubiquitin-mediated degradation. (40, 41). In parallel, Grimm et al. (42) demonstrated that multiple metabolic enzyme systems, including GAPDH, prime cells in normoxia for rapid HIF-1α stabilization upon inflammatory challenge (42). In LPS/IFN-γ-activated microglia, HIF-1α reciprocally transactivates Hk2, Pfkfb3, Pkm2, and Ldha, thereby reinforcing the glycolytic program initiated by SIRT1-dependent GAPDH deacetylation (43, 44). This feed-forward loop provides a molecular explanation for the pronounced glycolytic and pro-inflammatory phenotype of K254R microglia observed in our study and supports the emerging concept that post-translational activation of a single glycolytic enzyme can act as a rheostat for the entire HIF-1α-driven immunometabolic program (45). Future studies employing HIF-1α knockdown in K254R microglia will be required to formally dissect the hierarchical relationship between GAPDH K254 deacetylation and HIF-1α-mediated transcriptional amplification.

Another consideration is the dual nature of microglial glycolysis: HK2 ablation paradoxically enhances ischemic neuroinflammation through mitochondrial ROS accumulation, whereas HK2 is necessary for surveillance under homeostasis (2). Therefore, treating microglial glycolysis as a uniform pathology is unwarranted. Our acute LPS/IFN-γ model may not reflect the metabolic landscape of chronic neurodegeneration, where SIRT1 progressively declines with age and CD38-driven NAD_+_ depletion (46). This may underlie the impaired phagocytic capacity observed in aged microglia (35).

A key mechanistic question raised by these results is whether the amplified inflammatory output of K254R microglia is strictly cell-autonomous or propagated to neighboring cells through secreted metabolites and DAMPs. The conditioned media and Transwell experiments reported in section 3.6 indicate that both components operate concurrently: direct expression of GAPDH-K254R in a given cell is sufficient to drive cell-autonomous glycolytic inflammatory activation, but a substantial fraction (~55–65%) of the phenotype is transferable to naive microglia through cell-free supernatants and non-contact co-culture. Selective depletion of L-lactate, HMGB1, or extracellular ATP each attenuated a distinct arm of the recipient response, and their combined removal reduced recipient signaling to K254Q-level. This identifies a composite paracrine circuit in which L-lactate stabilizes HIF-1α and promotes histone-lactylation-driven pro-inflammatory transcription in bystander cells (40, 47), HMGB1, released via active, non-necrotic secretion, engages TLR4 (48, 49), and extracellular ATP amplifies IL-1β maturation through P2X7–NLRP3 (50). This model is consistent with recent evidence that microglia-derived metabolites and DAMPs collectively reprogram the metabolic and inflammatory state of neighboring glia (51, 52), It also explains why *in vivo* perturbation of a single microglial enzyme can generate field-scale inflammatory phenotypes despite incomplete penetrance of the primary lesion. Practically, this dissection identifies MCT1/4, P2X7, and HMGB1–TLR4 as candidate targets for restricting the propagation of SIRT1–GAPDH-driven neuroinflammation without ablating microglial surveillance functions.

### Limitations

Experiments using BV-2 cells and short-term primary cultures under a single inflammatory stimulus have not established whether the SIRT1–GAPDH axis operates identically *in vivo*, in aged microglia, or under chronic low-grade stimulation. Partial endogenous GAPDH replacement (~75–80%) means that residual WT protein may have moderated phenotypic differences. SRT1720 and EX-527 carry off-target liabilities, and definitive attribution requires microglia-specific Sirt1 conditional knockout or catalytic-dead knock-in models. The present study addresses the cell-autonomous versus paracrine question through conditioned media transfer, Transwell co-culture, and direct quantification of extracellular DAMPs (section 3.6), and identifies a mixed model in which cell-autonomous GAPDH-K254 deacetylation initiates the phenotype and secreted L-lactate, HMGB1, and ATP propagate it to neighboring microglia. Full *in vivo* validation of the relative contributions of these arms in aged or chronically inflamed brain remains to be established.

We further acknowledge that the ~75–80% siRNA-mediated replacement of endogenous GAPDH, although internally consistent across replicates, is intrinsically an overexpression paradigm in which residual WT GAPDH continues to occupy a fraction of the cellular pool. Although our linear-mixture dilution model (2.5) indicates that this residual pool slightly compresses—but does not qualitatively distort—the K254R/K254Q phenotype, definitive resolution requires endogenous CRISPR/Cas9 knock-in of the K254Q and K254R substitutions at the native murine GAPDH locus, an approach we are now pursuing (19, 20). A second consideration is that changes in NAD_+_ availability can modulate SIRT1 activity independently of SIRT1 protein levels. The newly incorporated NAD_+_/NADH measurements (3.1) demonstrate that a ~38% fall in the cytosolic NAD_+_/NADH ratio during LPS/IFN-γ activation contributes to the observed loss of SIRT1 deacetylase function, and that restoring NAD_+_ with nicotinamide riboside partially uncouples SIRT1 activity from SIRT1 protein abundance (46, 53). This dual regulation—decreased SIRT1 protein and decreased cofactor availability—jointly gates GAPDH K254 deacetylation under inflammatory stress.

Importantly, although pharmacological activation/inhibition and SIRT1 depletion support pathway involvement, an siRNA-resistant SIRT1 overexpression rescue is required to establish whether restoring SIRT1 abundance during the 24-h inflammatory state reverses GAPDH K254 acetylation, glycolytic flux, and cytokine output. Such a rescue is particularly important because the late reduction in endogenous SIRT1 constrains interpretation of additional siRNA-mediated depletion. Most importantly, all mechanistic experiments were performed in BV-2 cells and short-term primary microglia under acute LPS/IFN-γ stimulation, and the physiological relevance of the SIRT1–GAPDH K254 axis in chronic neuroinflammation—for example in experimental autoimmune encephalomyelitis (EAE), 5×FAD Alzheimer’s disease, and MPTP PD models—therefore remains unresolved. In these disease contexts, microglia are exposed to sustained, spatially heterogeneous, and often lower-grade inflammatory cues, and NAD_+_ availability progressively declines with age and CD38 up-regulation, both of which could quantitatively or qualitatively alter the SIRT1–GAPDH set-point observed in the present *in vitro* system (34, 54). Definitive *in vivo* validation therefore requires cell-type-specific genetic tools rather than pharmacological modulators alone.

### Future directions

The next priority is to express an siRNA-resistant WT SIRT1 construct, alongside a catalytically inactive mutant, in LPS/IFN-γ-treated BV-2 cells and primary microglia. This gain-of-function rescue will let us quantify total SIRT1, GAPDH K254 acetylation, GAPDH activity, glycoPER, lactate, and cytokine release at 24 h, which should separate true restoration of SIRT1 abundance from nonspecific transfection artifacts. This approach fits with recent work showing that SIRT1’s control over glycolysis and microglial inflammatory phenotype depends heavily on context (28, 35). Beyond this, we plan microglia-specific Sirt1 conditional knockout studies in neurodegeneration models, along with a systematic map of how K254 acetylation interacts with competing GAPDH modifications. To establish whether the SIRT1–GAPDH K254 axis actually matters in chronic neuroinflammation *in vivo*, we propose two parallel genetic strategies. First, we will generate microglia-specific Sirt1 conditional knockouts by crossing Sirt1^fl/fl mice with the tamoxifen-inducible Cx3cr1-CreERT2 driver. A chase period of at least 28 days will follow tamoxifen induction to allow peripheral monocyte turnover, so that recombination is confined to long-lived, brain-resident microglia rather than infiltrating cells (54, 55). These mice will then be tested across three chronic disease models: MOG35–55-induced EAE, which captures adaptive-immunity-driven demyelination; the 5×FAD model of amyloid-β pathology; and sub-acute MPTP intoxication, which models dopaminergic neurodegeneration (28, 56, 57). Second, we will build a GAPDH K254R knock-in mouse using CRISPR/Cas9 homology-directed repair, placing a constitutively deacetylation-mimetic GAPDH at its endogenous locus (58). Our *in vitro* reconstitution tops out at roughly 75–80% replacement and always leaves some WT protein behind; a global knock-in avoids both problems and gives the strictest possible genetic test of whether K254 deacetylation actually drives microglial inflammatory glycolysis at the whole-organism level. We also plan a microglia-restricted version—GAPDH^lox-stop-lox-K254R crossed to Cx3cr1-CreERT2—as a cell-autonomous control.

These models generate three hypotheses we can directly test and, in principle, falsify. (i) Deleting Sirt1 specifically in microglia will, counterintuitively, dampen rather than worsen glycolytic priming and cytokine output in the EAE and MPTP models. Clinical EAE scores, dopaminergic neuron loss in the substantia nigra, and Iba1_+_/CD68_+_ microgliosis should all decrease, even as classical SIRT1-controlled transcriptional readouts—acetyl-p65, NF-κB target genes—go up. If this pans out, it will split SIRT1’s transcriptional and metabolic roles apart *in vivo*, showing they do not move in the same direction.

(ii) The GAPDH K254R knock-in should phenocopy SIRT1 activation: higher microglial glycolytic flux, elevated interstitial lactate, faster plaque-associated microgliosis in the 5×FAD background, and worse motor deficits after MPTP. Microglia-targeted 2-deoxy-D-glucose or PFKFB3 inhibition should reverse all of it (56, 59). (iii) In 5×FAD mice crossed with the Sirt1 conditional knockout, restoring NAD_+_ with nicotinamide riboside should rescue GAPDH K254 deacetylation and normalize cytokine output—but only in Cre-negative controls, not in mice lacking microglial SIRT1. This would pin down microglial SIRT1 as the essential link in the NAD_+_–GAPDH–inflammation chain, at least in a chronic amyloid setting.

Readouts across these experiments will include quantitative K254-acetyl GAPDH immunohistochemistry on FACS-purified CD11b_+_CD45^int microglia, single-cell RNA sequencing of the disease-associated microglia (DAM) signature, ¹³C-glucose flux analysis on freshly isolated microglia, and behavioral testing (EAE clinical scoring, Morris water maze, rotarod). Together, this work would take the mechanism we have established in cell culture and subject it to a genuinely rigorous *in vivo* test—and tell us whether the SIRT1–GAPDH K254 switch is a real, cell-type-selective target worth pursuing in chronic neuroinflammatory and neurodegenerative disease.

## 5 Conclusion

Microglial neuroinflammation is fueled by aerobic glycolysis; however, the post-translational switches that gate this flux have not been completely defined. The present study positions SIRT1-mediated GAPDH K254 deacetylation as a switch that is mechanistically distinct from SIRT1’s transcriptional substrates and operates directly on the enzymatic engine of glycolysis. Targeting the K254 mark, either pharmacologically or by manipulating NAD_+_ supply to SIRT1, offers a substrate-resolved alternative to broad sirtuin modulation in neuroinflammatory diseases.

## Data Availability

The data analyzed in this study is subject to the following licenses/restrictions: Data will be made available on request. Requests to access these datasets should be directed to Dr. Chou-Yi Hsu Email: t545316@gmail.com.
