# Supplementary material for: SIRT1 deacetylates GAPDH to drive microglial glycolysis and neuroinflammation
Source: Front Immunol. 2026 Aug 18;17:1883095. doi: 10.3389/fimmu.2026.1883095 (PMC13529471; doi:10.3389/fimmu.2026.1883095)
Supplement: Supplementary file 1 [file Supplementaryfile1.docx]

**Supplementary Figure S1.** Validation of SIRT1 knockdown and endogenous GAPDH replacement with K254 acetylation-state mutants in BV-2 microglia. (A) Representative immunoblots confirm SIRT1 depletion following transfection with SIRT1-targeting siRNA (siSIRT1) relative to non-targeting (NT) siRNA. β-Actin served as the loading control. Three independent lysates are shown per condition. (B) Densitometric quantification of SIRT1 protein abundance, normalized to β-actin and expressed as a percentage of the NT-siRNA control, shows that siSIRT1 significantly reduced SIRT1 protein expression (P < 0.01; n = 4 independent biological replicates). (C) Representative immunoblots validate the GAPDH replacement strategy. Endogenous murine Gapdh was depleted using a 3'-UTR-targeting siRNA (siGapdh), followed by transfection with either an empty vector or FLAG-tagged human GAPDH K254Q (acetylation-mimetic) or K254R (deacetylation-mimetic) constructs. Anti-FLAG (M2) detected exogenous human GAPDH, while anti-GAPDH detected both endogenous murine and exogenous human GAPDH. Mock-transfected cells treated with NT siRNA served as the control. (D) Replacement efficiency, calculated as FLAG-GAPDH/(FLAG-GAPDH + endogenous GAPDH) × 100, was comparable between K254Q and K254R constructs and exceeded the predefined 75% cutoff (dashed line). Data are mean ± SEM from n = 5 independent biological replicates; each circle represents one biological replicate. n.s., not significant.
