# Supplementary material for: SIRT1 deacetylates GAPDH to drive microglial glycolysis and neuroinflammation
Source: Front Immunol. 2026 Aug 18;17:1883095. doi: 10.3389/fimmu.2026.1883095 (PMC13529471; doi:10.3389/fimmu.2026.1883095)
Supplement: Supplementary file 2 [file Supplementaryfile2.docx]

**Supplementary Figure S2.** Re-probing of the pan-acetyl-lysine membrane with a site-specific anti-acetyl-GAPDH-K254 antibody confirms comparable basal K254 acetylation in wild-type and scramble-siRNA controls and documents an exploratory SIRT1-siRNA result in BV-2 microglia. (A) Representatives immunoblot of the PVDF membrane originally used for the pan-acetyl-lysine analysis in Figure 2E, stripped and re-probed with anti-acetyl-K254 antibody (Abcam, ab280577), together with GAPDH and β-actin loading controls. Whole-cell lysates (25 µg per lane) were loaded as follows: unstimulated wild-type (WT), LPS/IFN-γ-stimulated WT, resting scramble-siRNA, LPS/IFN-γ-stimulated scramble-siRNA, and LPS/IFN-γ-stimulated SIRT1-siRNA. Ac-K254 immunoreactivity was comparably low in unstimulated WT and resting scramble-siRNA samples and increased after LPS/IFN-γ stimulation. In this re-probing experiment, the stimulated SIRT1-siRNA lane showed lower Ac-K254 signal than the stimulated scramble-siRNA lane. (B) Densitometric quantification of Ac-K254 normalized to total GAPDH. Bars show mean ± SEM with individual biological replicates (n = 4). Unstimulated WT and resting scramble-siRNA groups did not differ (p = 0.72, unpaired two-tailed t-test), indicating that scramble transfection did not alter basal K254 acetylation. The lower Ac-K254 signal in the stimulated SIRT1-siRNA group relative to stimulated scramble-siRNA was significant (p < 0.001). Because this direction is not consistent with a simple loss-of-deacetylase model, the siRNA comparison is considered exploratory and is not used to establish the catalytic direction of SIRT1-mediated K254 regulation; independent replication with validated knockdown, rescue, and orthogonal site-quantification is required.
