# Supplementary material for: SIRT1 deacetylates GAPDH to drive microglial glycolysis and neuroinflammation
Source: Front Immunol. 2026 Aug 18;17:1883095. doi: 10.3389/fimmu.2026.1883095 (PMC13529471; doi:10.3389/fimmu.2026.1883095)
Supplement: Supplementary file 3 [file Supplementaryfile3.docx]

**Supplementary Figure S3.** Ancillary short-term primary-microglia experiment: NAD⁺/NADH ratios and SIRT1 deacetylase activity.

Primary murine microglia (>95% CD11b⁺) were untreated or stimulated with LPS (100 ng/mL) plus IFN-γ (20 ng/mL) for 6 h. Where indicated, cells received nicotinamide riboside (NR, 500 µM), FK866 (10 nM), EX-527 (10 µM), or resveratrol (RSV, 25 µM). This experiment differs from the main 24-h BV-2/primary-microglia experiment in stimulus duration, IFN-γ concentration, pharmacological activator, and sample size; it should therefore be interpreted as ancillary pharmacological evidence and not as a direct numerical replicate of the main-text dataset. (A) Intracellular NAD⁺/NADH ratio measured by colorimetric assay (Sigma MAK037). (B) SIRT1 deacetylase activity measured by fluorometric assay (Sigma MAK277), expressed as a percentage of basal. (C) Regression of SIRT1 activity against NAD⁺/NADH ratio for the physiological conditions (Basal, LPS/IFN-γ, LPS+NR, and LPS+FK866); direct pharmacological modulators were excluded because they can alter SIRT1 activity independently of NAD⁺ availability. (D) Parallel changes in NAD⁺/NADH ratio and Ac-K254-GAPDH in the NR/FK866 experiment. Data are mean ± SEM, n = 6 independent biological replicates per condition, pooled from three experiments. One-way ANOVA with Tukey’s post-hoc test was used for group comparisons. The results support cofactor sensitivity of SIRT1 activity under these conditions but do not establish the full causal mechanism or replace direct replication under the primary manuscript conditions.
