# Supplementary material for: SIRT1 deacetylates GAPDH to drive microglial glycolysis and neuroinflammation
Source: Front Immunol. 2026 Aug 18;17:1883095. doi: 10.3389/fimmu.2026.1883095 (PMC13529471; doi:10.3389/fimmu.2026.1883095)
Supplement: Supplementary file 4 [file Supplementaryfile4.docx]

**Supplementary Figure S4.** IL-10 secretion is not altered by GAPDH K254 acetylation-state mimetics in activated microglia. BV-2 microglia (A) and primary microglia isolated from postnatal day 3–5 C57BL/6J mice (B) expressing wild-type (WT), acetylation-mimetic K254Q, or deacetylation-mimetic K254R GAPDH following endogenous Gapdh knockdown were stimulated with LPS (100 ng/mL) and IFN-γ (10 ng/mL) for 24 h. IL-10 concentrations in conditioned media were quantified by ProcartaPlex multiplex assay. Bars represent mean ± SEM; individual dots represent independent biological replicates (BV-2, n = 5; primary microglia, n = 4). Statistical analysis was performed using one-way ANOVA followed by Tukey’s multiple-comparisons test. No pairwise comparison was statistically significant (ns, not significant).
