# Supplementary material for: SIRT1 deacetylates GAPDH to drive microglial glycolysis and neuroinflammation
Source: Front Immunol. 2026 Aug 18;17:1883095. doi: 10.3389/fimmu.2026.1883095 (PMC13529471; doi:10.3389/fimmu.2026.1883095)
Supplement: Supplementary file 5 [file Table1.docx]

# Supplementary Table S1. Statistical Analysis Summary: Tests Used, Sample Sizes, and Power Calculations

| **#** | **Outcome / Experiment** | **Data Type** | **Groups** | **Statistical Test** | **Post-hoc** | **Normality** | **n (biol.)** | **Tech. Reps.** | **Threshold** |
| --- | --- | --- | --- | --- | --- | --- | --- | --- | --- |
| 1 | GAPDH K254 acetylation (Control vs LPS/IFN-γ) | Continuous, parametric | 2 | Two-tailed t-test | n/a | Shapiro–Wilk | ≥ 3 | Duplicate WB lanes | p < 0.05/0.01/0.001 |
| 2 | SIRT1–GAPDH Co-IP stoichiometry | Paired continuous | 2 (paired) | Paired t-test | n/a | Shapiro–Wilk | ≥ 3 | 2 blots/run | p < 0.05 |
| 3 | Lactate concentration (K254Q vs K254R) | Continuous, parametric | 2 | Two-tailed t-test | n/a | Shapiro–Wilk | ≥ 3 | Duplicate wells | p < 0.01 |
| 4 | qRT-PCR (Tnfa, Il1b, Nos2: K254Q vs K254R) | Continuous (2⁻ΔΔCt) | 2 | Two-tailed t-test | n/a | Shapiro–Wilk | ≥ 3 | Triplicate qPCR wells | p < 0.05 |
| 5 | Nitric oxide (Griess; K254Q vs K254R) | Continuous, parametric | 2 | Two-tailed t-test | n/a | Shapiro–Wilk | ≥ 3 | Duplicate wells | p < 0.01 |
| 6 | Non-normal two-group fallback | Continuous, non-parametric | 2 | Mann–Whitney U | n/a | Shapiro–Wilk failed | ≥ 3 | — | p < 0.05 |
| 7 | SIRT1 protein over time (0, 6, 24 h) | Continuous, parametric | 3 | One-way ANOVA | Tukey's HSD | Shapiro–Wilk | ≥ 3 | Duplicate blots | p < 0.05/0.01 |
| 8 | GAPDH K254 across pharmacology | Continuous, parametric | ≥ 3 | One-way ANOVA | Tukey's HSD | Shapiro–Wilk | ≥ 3 | Duplicate WB | p < 0.001 |
| 9 | GAPDH enzymatic activity (WT/K254Q/K254R) | Continuous, parametric | 3 | One-way ANOVA | Tukey's HSD | Shapiro–Wilk | ≥ 3 | Duplicate kinetic reads | p < 0.001 |
| 10 | Multiplex ELISA cytokines | Continuous, parametric | ≥ 3 | One-way ANOVA | Tukey's HSD | Shapiro–Wilk | ≥ 3 | Duplicate ProcartaPlex | p < 0.01 |
| 11 | SIRT1 NAD⁺ deacetylase (Time × Treatment) | Continuous, parametric | Factorial 2×k | Two-way ANOVA | Bonferroni | Shapiro–Wilk | ≥ 3 | Duplicate fluorometric | Interaction p < 0.05 |
| 12 | Seahorse glycoPER (Genotype × Stimulation) | Continuous, parametric | Factorial 2×2 | Two-way ANOVA | Bonferroni | Shapiro–Wilk | ≥ 3 | ≥ 5 wells/condition (XFe96) | Interaction p < 0.05 |
| 13 | Correlation SIRT1 ↔ GAPDH activity | Bivariate continuous | Paired obs. | Pearson's r | n/a | Shapiro–Wilk (both axes) | ≥ 3 | — | r = 0.71; p < 0.001; 95% CI |
| 14 | A priori sample size justification | Power analysis | — | G*Power-style | n/a | n/a | Determined ≥ 3 | — | Power=0.80; α=0.05; pilot ES |
| 15 | Cytokine LLOQ handling | Detection censoring | — | Values < LLOQ excluded | n/a | n/a | — | — | LLOQ per ProcartaPlex |
| 16 | Western blot densitometry | Continuous, normalized | — | Per-panel t-test/ANOVA | Tukey / Bonferroni | Shapiro–Wilk | ≥ 3 | 2 lanes per blot | p < 0.05 |

Comprehensive summary of every statistical procedure applied in the study, mapping each test to its specific experimental outcome, with replicate structure, power parameters, normalization strategy, and software details. All analyses were performed in GraphPad Prism v10.0; α = 0.05; results reported as mean ± SEM; n ≥ 3 independent biological replicates conducted on separate days.
