# Supplementary material for: SIRT1 deacetylates GAPDH to drive microglial glycolysis and neuroinflammation
Source: Front Immunol. 2026 Aug 18;17:1883095. doi: 10.3389/fimmu.2026.1883095 (PMC13529471; doi:10.3389/fimmu.2026.1883095)
Supplement: Supplementary file 6 [file Table2.docx]

**Supplementary Table S2.** Dilution-corrected intrinsic activity and flux values for GAPDH K254 mutants.

Enzymatic activity, glycolytic flux (glycoPER), lactate output, and pro-inflammatory cytokine levels were measured in primary murine microglia expressing FLAG-tagged human GAPDH-WT, K254R (deacetylation-mimetic), or K254Q (acetylation-mimetic) following 3'-UTR siRNA knockdown of endogenous Gapdh. Given that knockdown efficiency was approximately 75–80% (Fig. S1), residual endogenous WT GAPDH (mean fraction f_WT = 0.225) contributed to the observed signal. To account for this, a linear-mixture model was applied: X_obs = f_WT · X_WT + (1 − f_WT) · X_mut. This equation was solved for the intrinsic mutant value, X_mut = (X_obs − f_WT · X_WT) / (1 − f_WT). Standard error propagation was utilized for the standard error of the mean (SEM). Data are presented as mean ± SEM, with n = 6 biological replicates per group, pooled from three independent experiments. LPS/IFN-γ challenge conditions were 100 ng mL⁻¹ + 20 ng mL⁻¹, respectively, for 6 hours.

| **Parameter** | **Condition** | **WT observed** | **K254R observed** | **K254R correctedᵃ** | **K254Q observed** | **K254Q correctedᵃ** | **Pᵇ** |
| --- | --- | --- | --- | --- | --- | --- | --- |
| GAPDH activity (µmol NADH min⁻¹ mg⁻¹) | Basal | 4.82 ± 0.21 | 5.14 ± 0.24 | **5.24 ± 0.28** | 4.36 ± 0.19 | **4.23 ± 0.22** | 0.041 |
|  | LPS/IFN-γ | 8.91 ± 0.34 | 10.62 ± 0.41 | **11.12 ± 0.48** | 6.28 ± 0.27 | **5.51 ± 0.31** | <0.001 |
| glycoPER (pmol min⁻¹ per 10⁴ cells) | Basal | 42.1 ± 2.3 | 46.7 ± 2.6 | **48.0 ± 3.1** | 36.4 ± 2.1 | **34.8 ± 2.4** | 0.008 |
|  | LPS/IFN-γ | 128.4 ± 5.2 | 156.9 ± 6.8 | **165.2 ± 7.4** | 84.7 ± 4.3 | **72.0 ± 4.9** | <0.001 |
| Lactate (nmol h⁻¹ per 10⁵ cells) | Basal | 18.6 ± 1.1 | 21.4 ± 1.3 | **22.2 ± 1.6** | 14.8 ± 0.9 | **13.7 ± 1.1** | 0.003 |
|  | LPS/IFN-γ | 57.3 ± 2.4 | 71.8 ± 3.1 | **76.0 ± 3.6** | 36.9 ± 1.9 | **31.0 ± 2.2** | <0.001 |
| TNF-α (pg mL⁻¹) | Basal | 32 ± 5 | 41 ± 6 | **43.6 ± 7.3** | 24 ± 4 | **21.7 ± 4.6** | 0.019 |
|  | LPS/IFN-γ | 1284 ± 62 | 1642 ± 78 | **1746 ± 92** | 742 ± 41 | **585 ± 48** | <0.001 |
| IL-6 (pg mL⁻¹) | Basal | 18 ± 3 | 24 ± 4 | **25.7 ± 4.8** | 13 ± 2 | **11.5 ± 2.3** | 0.024 |
|  | LPS/IFN-γ | 896 ± 44 | 1148 ± 58 | **1221 ± 66** | 521 ± 31 | **412 ± 36** | <0.001 |
| IL-1β (pg mL⁻¹) | Basal | 12 ± 2 | 15 ± 3 | **15.9 ± 3.4** | 9 ± 1 | **8.1 ± 1.2** | 0.036 |
|  | LPS/IFN-γ | 642 ± 32 | 824 ± 41 | **877 ± 47** | 368 ± 22 | **290 ± 25** | <0.001 |

ᵃ Corrected mutant value from the linear-mixture model: X_mut = (X_obs − f_WT·X_WT) / (1 − f_WT), with f_WT = 0.225 (mean residual endogenous GAPDH fraction, Fig. S1). SEM was propagated by standard error propagation.

ᵇ Two-way ANOVA (genotype × stimulation) with Tukey's HSD post-hoc; P value shown for K254R vs. K254Q corrected means within the same stimulation condition.

Abbreviations: glycoPER, glycolytic proton efflux rate; LPS, lipopolysaccharide; IFN-γ, interferon-gamma; WT, wild-type; K254R, deacetyl-mimetic; K254Q, acetyl-mimetic; SEM, standard error of the mean.
